# La Palma island (Spain) geothermal system revealed by 3D magnetotelluric data inversion

**DOI:** 10.1038/s41598-020-75001-z

**Published:** 2020-10-23

**Authors:** Federico Di Paolo, Juanjo Ledo, Katarzyna Ślęzak, David Martínez van Dorth, Iván Cabrera-Pérez, Nemesio M. Pérez

**Affiliations:** 1 Instituto Volcanológico de Canarias (INVOLCAN), 38200 San Cristóbal de La Laguna, Spain; 2 Departament de Dinàmica de la Terra i de l’Oceà, Universitat de Barcelona, 08028 Barcelona, Spain; 3 Instituto Tecnológico y de Energías Renovables (ITER), 38600 Granadilla de Abona, Spain; 4 Agencia Insular de la Energía de Tenerife (AIET), 38600 Granadilla de Abona, Spain; 5 Present Address: Dipartimento di Scienze e Tecnologie, Università degli Studi di Napoli “Parthenope”, 80143 Naples, Italy; 6 Present Address: Departamento de Geofísica, Facultad de Ciencias Físicas y Matemáticas, Universidad de Chile, Santiago, Chile

**Keywords:** Volcanology, Geophysics

## Abstract

The study of geothermal systems is nowadays a topic of great importance because of the huge amount of energy that could be converted in electricity for human consumption from such sources. Among the various geophysical methods employed to study geothermal reservoirs, the magnetotelluric (MT) method is capable to reveal the internal structures of the subsurface and interpret the geological structures from the electrical resistivity. We present the first 3D resistivity model of La Palma (Canary archipelago, Spain) obtained from a dataset of 44 broadband magnetotelluric soundings distributed around the island. Our results highlight the presence of resistivity anomalies, spatially coinciding with density anomalies present in literature. In the north of the island, a high resistivity anomaly can be interpreted as the signature of an old intrusive body beneath the Taburiente caldera. In the south, a complex resistivity structure around the Cumbre Vieja volcanic ridge could be indicative of presence of an active geothermal system. In particular, low-resistivity anomalies, located in a high-fractured zone, have values compatible with clay alteration caps (illite and illite–smectite). Such a result suggests the presence of hot rocks, or a dike system, heating fluids in the interior of Cumbre Vieja volcanic system.

## Introduction

The use of geophysics to map the presence of magmatic/geothermal reservoirs beneath the surface of volcanic islands has been successfully employed not just for scientific purposes but also as a first step to evaluate the possibility of exploration and exploitation of the geothermal resources^1^. As a matter of fact, in an isolated archipelago like the Canary Islands, the geothermal exploitation could provide a huge amount of energy convertible in electricity for human consumption since medium- to high-temperature resources, which are usually linked to active volcanic regions, are needed for it^2^.

The Canary Islands are an intraplate volcanic archipelago comprising seven main islands and four islets located in the eastern Atlantic Ocean, between latitudes 27° N and 30° N. The islands are aligned in the E–W direction, extending about 500 km, with the most eastern one (Fuerteventura) being ~ 100 km away from NW Africa. The volcanic activity of the archipelago started in Oligocene and it is still in progress^3^. The origin of volcanism and the geodynamic evolution of the archipelago are still under debate. After some competing models such as a propagating fracture from the Atlas Mountains^4^, intraplate alkaline volcanism associated with fractures^5^ or the presence of a mantle plume or hotspot beneath the Canaries^6–8^, Anguita and Hernán^9^ proposed a unifying model considering the co-existence of a thermal anomaly in the mantle connected to tectonic mechanisms in a regionally fractured area.

**Figure 1:**
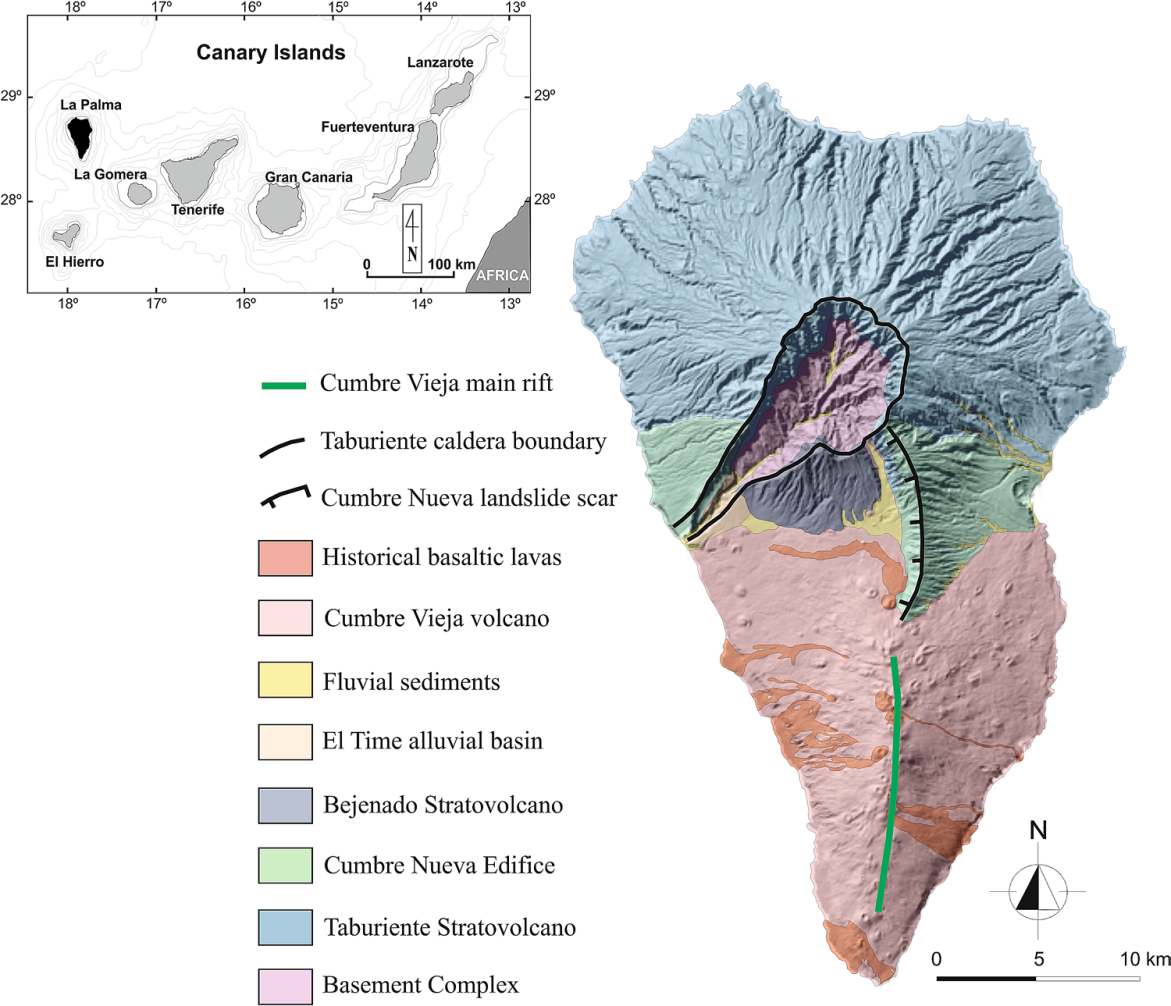
Geological map of La Palma island (after Padrón et al.^15^).

Located in the western part of the archipelago, La Palma is one of the youngest islands, the second in height and the fifth largest in area of 706 km^2^, and is elongated in the N–S direction, with a length of about 45 km. The geological evolution of the island, connected to the most significant visible volcanic edifices, can be summarized as^10^: (i) the old basal complex (ca. 4 to 3 Ma) comprising a Pliocene seamount sequence and a plutonic complex, uplifted and tilted by subsequent intrusions, currently outcropping only inside the Taburiente caldera thanks to an extensive erosion; (ii) a volcanic series (1.7 to 0.4 Ma), including the Garafía volcano, the Taburiente shield volcano and Cumbre Nueva and Bejenado edifices, covering the northern part (more than a half in surface) of the island; and (iii) the Cumbre Vieja series (123 ka to present), a ridge system having rift, faults and volcanic vents aligned along its N–S crest and whose volcanic products cover the southern part of the island. Such volcanic units are shown in Fig. 1: also visible are the depressions of the Cumbre Nueva landslide and the Taburiente caldera (both originated from lateral collapses and subsequently enlarged by intense erosion^11^), and the Cumbre Vieja ridge.

During the last 500 years Cumbre Vieja, in the southern part of the island, has experienced at least six eruptions^11^, with the last eruptive episodes occurring in 1971 (Teneguía volcano). Associated to the main N–S rift, a system of normal faults usually parallel but occasionally oblique to the rift axis has developed. As pointed out by Galipp et al.^12^, Cumbre Vieja has probably been fed by a distinct magma reservoir/plumbing system with respect to the older Taburiente/Cumbre Nueva volcanic system. Such a distinction in between the two volcanic complexes has been also evidenced by Camacho et al.^13^ from the interpretation of gravity anomalies in the interior of the island. Furthermore, a shallow intrusive sills and dikes complex has been suggested to be present beneath Cumbre Vieja volcano^14^.

As a consequence of recent volcanic activity, the presence of an active geothermal system with associated passive degassing of volcanic gases along the N–S rift zone of Cumbre Vieja and its fault system is well documented^14–16^. High soil temperatures (90–130 °C) have recently been measured during a high CO_2_ emission period^16^. Furthermore, in the south of the island a hot spring complex (Fuente Santa), that had been completely covered by lava in 1677^11^ but rediscovered in 2005^17^, exhibits temperatures above 40 °C, and ${\mathrm{HCO}}_{3}^{-}$ and ${\mathrm{SO}}_{4}^{2-}$ concentrations exceeding 2000 mg/L^15^. Such a composition in the water can be related to groundwater circulation: due to the dikes’ geometry the circulating meteoric water follows the main structural N–S rift-zone of Cumbre Vieja, from the recharge area towards the south of the island, experiencing heating and contamination due to the presence of volcanic gases such as CO_2_, SO_2_ and H_2_S.

The use of MT to map the internal structure of volcanic/geothermal areas is well documented, both for the assessment of the geothermal potential and the connected hydrothermal circulation, and for structural investigation and magmatic reservoir characterization^1,18^. In particular, a 3D resistivity model of the subsurface of a volcanic-geothermal area surveyed by MT can reveal the presence and the shape of such structures^19–30^. Volcanic geothermal areas are usually characterized by a low resistivity clay-cap layer associated with the presence of smectite (< 10 Ω⋅m) and illite–smectite (up to a few tens of Ω⋅m) overlying a resistive zone where the reservoir is located, and such a sharp contrast can be easily detected by the application of MT^1^.

Here we present the first 3D resistivity model of La Palma island (Canary archipelago, Spain) obtained by a broadband MT survey of the entire island consisting of 44 sites of observation. Up to now, the only EM survey performed in La Palma has been reported by García and Jones^31^, which presented three 2D MT sections in the western part of the island and a synthetic 3D study of the coast effect on their MT data.

Between June and August 2018, 44 broadband MT stations have been deployed on La Palma island (Supplementary Fig. S1). The instrumentation consisted of four Metronix ADU-08e equipped with EPF-06 electrodes and MFS-06e magnetic coils. At each site the horizontal components of the electromagnetic field (${E}_{x}$, ${E}_{y}$, ${H}_{x}$ and ${H}_{y}$) were recorded, orienting the x-axis along the N–S direction pointing the north and y-axis in the E–W direction pointing the east. The vertical component of the magnetic field has not been measured due to the highly compact and hard soil in the majority of the measurement points. To improve the data quality two reference stations were placed in order to remove uncorrelated noise^32,33^.

## Inversion and results

The 3D electrical resistivity model has been obtained from the inversion of the four components of impedance tensor using the ModEM code^34^, an EM inversion and modeling program based on a standard minimum-structure, non-linear conjugate gradients algorithm. Full tensor components at 17 periods in the range 10^–3^ to 10^3^ s were used, with a resistivity model consisting of 89 × 121 horizontal and 84 vertical layered-grid (with a minimum cell size of 400 m in the horizontal directions and 100 m vertical cell size for the subaerial part of the island, and 10 m at sea level, increasing by a factor 1.2 to depth) in which the topography, the surrounding bathymetry of the island and the seawater resistivity (0.3 Ω⋅m) have been included. The initial model was set to a background resistivity of 100 Ω⋅m. It is important to note that La Palma island is characterized by a very steep topography which, together with the surrounding ocean, has an impact on the observed MT responses. Therefore, as suggested by Piña-Varas et al.^24^ the effects of the conductive surrounding ocean and the seafloor topography have been included in order to obtain a reliable resistivity structure in the interior of the island. The initial normalized RMS was 20 and the final one 1.97 after 91 iterations using and error floor of 5% for the off-diagonal impedance components and a 10% error floor for the diagonal impedance components. Supplementary Fig. S5 show the error floor distribution for each site.

**Figure 2:**
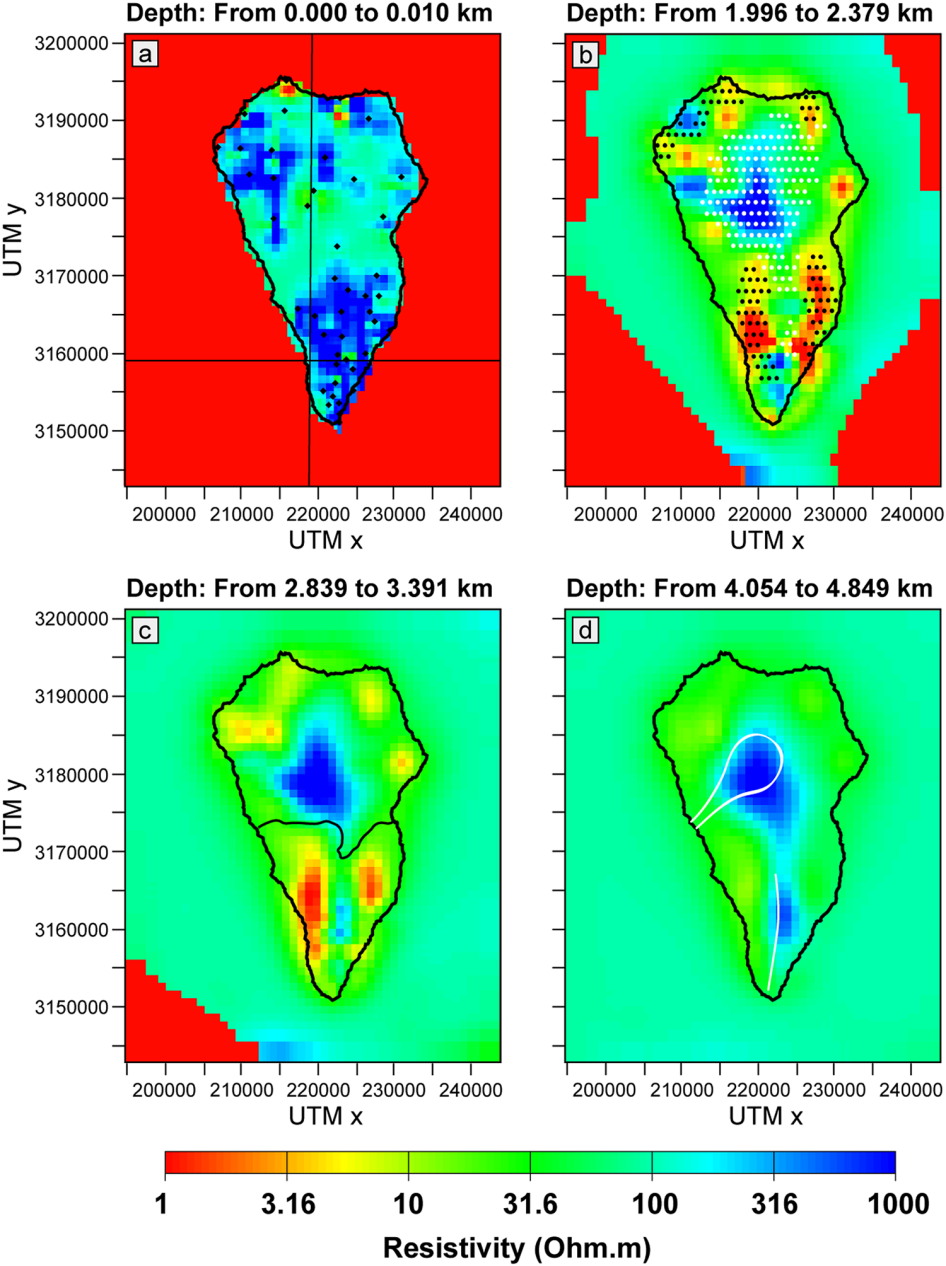
Horizontal sections from the 3D resistivity model at different depths. (**a**) 0 km (sea level); black diamonds represent the positions of the MT station; black lines represent the positions of the sections reported in Figs. 3, 4. (**b**) 2 km; white and black pointed textures represent the high and low (respectively) density anomalies reported by Prieto et al.^35^ at 2 km depth. (**c**) 3 km; black solid line represents the limit of Cumbre Vieja^10^. (**d**) 4 km; white lines represent the Taburiente caldera rim and the Cumbre Vieja ridge in the northern and southern parts of the island, respectively.

**Figure 3:**
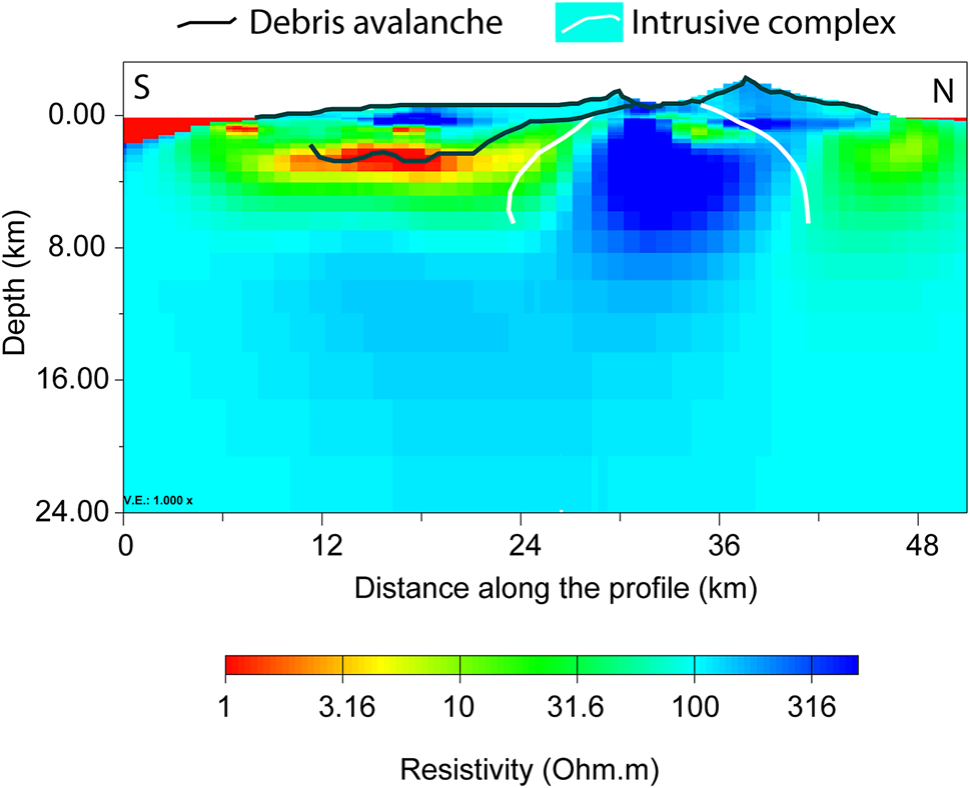
Vertical N–S section (x = 219,000 UTM) from the 3D resistivity model. Bold black line, black line and white line represent island topography, debris/sedimentary deposit limit and intrusive complex limits, respectively, as proposed by Gonzalez et al.^36^.

**Figure 4:**
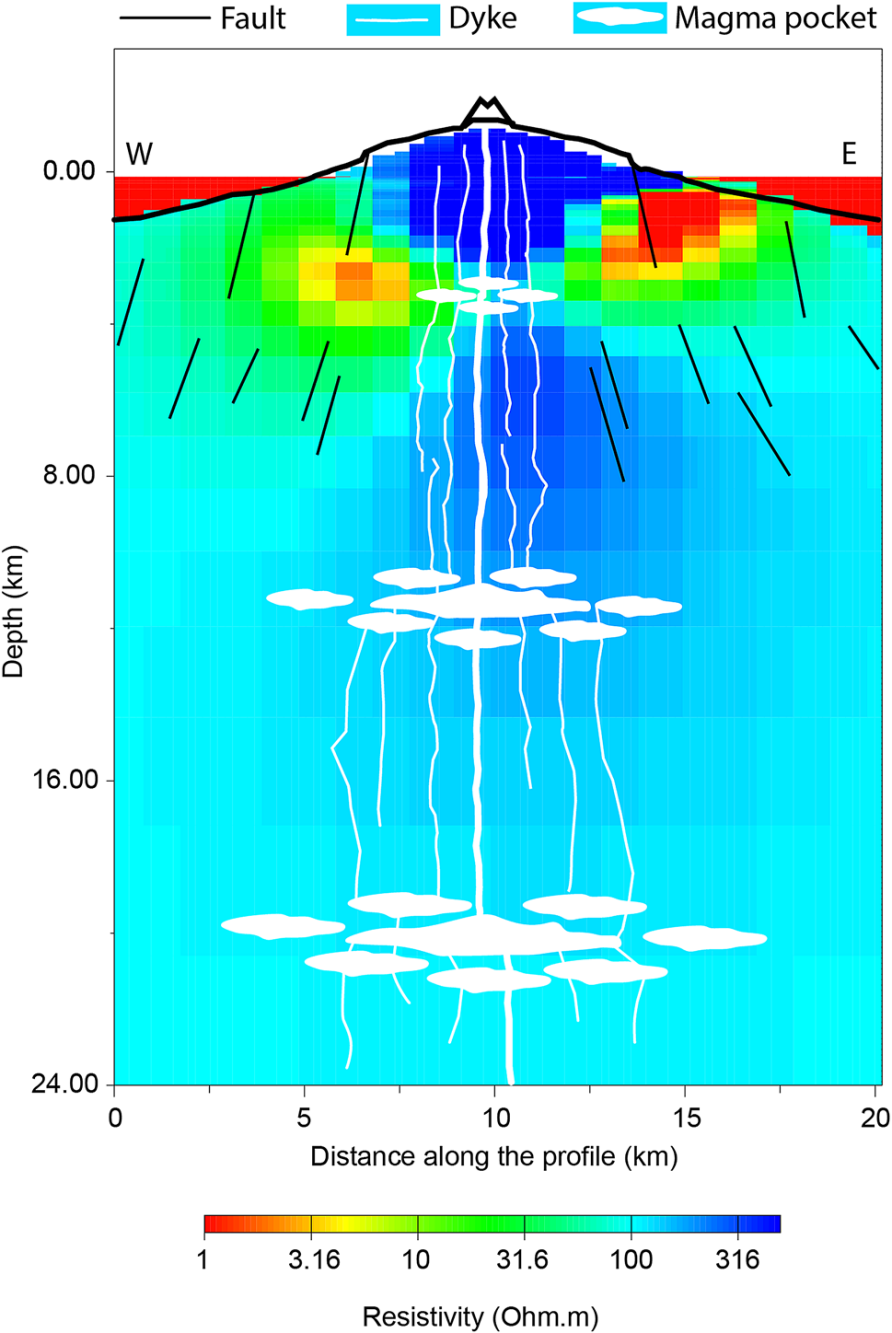
Vertical E–W section (y = 3,159,000 UTM) from the 3D resistivity model. Bold black line, black lines, white lines and white blobs represent island topography, faults, dikes, and magma pockets, respectively, as proposed by Klügel et al.^14^.

Figure 2 reports four plan view sections of our 3D resistivity model of La Palma around the depths of 0 (sea level), 2, 3 and 4 km below sea level. In Fig. 2a the positions of our MT stations (black diamonds) and the position of the vertical cross sections reported in Figs. 3, 4 (black lines) are evidenced. In Fig. 2b the white and black pointed textures represent the high- and low-density anomalies (respectively) reported by Prieto et al.^35^ at 2 km of depth. In Fig. 2c the black line represents the northern limit of Cumbre Vieja^10^. In Fig. 2d the Taburiente caldera rim and the Cumbre Vieja ridge are evidenced with white lines. At sea level (Fig. 2a) the resistivity values inside the island are quite homogeneous without strong lateral gradients. Only a diffused high resistivity structure is imaged in the southern part of the island. At 2 km b.s.l. (Fig. 2b) a generalized decrease of the resistivity is observed with a few scattered low resistivity structures (1–3 Ω⋅m) appearing in the eastern and northern coast of the island. The most interesting and spatially extended features are revealed at a depth of 2–3 km below the sea level (Figs. 2b,c). The main resistive body is noticeable in the center of the northern part of the island, beneath and around the Taburiente caldera, where the older volcanic edifices are located. On the other hand, the main conductive structures, having a N–S elongation, are located in the southern part of the island around the Cumbre Vieja ridge, the actually active volcanic system of the island. In between these two conductive zones, a more resistive body having the same elongation is also present (Fig. 2d).

Two vertical sections of the 3D resistivity model are reported in Figs. 3 and 4. In Fig. 3, in order to help the interpretation of resistivity values, the N–S geological units proposed by Gonzalez et al.^36^ are reported: the debris avalanche deposit or sedimentary apron on the western flank of Cumbre Vieja in black, and the limits of the intrusive complex beneath Taburiente caldera in white. The same resistivity anomalies emerging from Fig. 2 are evident also in Fig. 3. Figure 4 (along latitude y = 3,159,000 UTM) is an E–W section located in the central part of Cumbre Vieja, in the south of the island. It is evident the presence of a high resistivity complex at surface, along the mountain ridge, the two conductive layers around it at depth and, in the deep central part, a medium–high resistivity complex. To highlight our results, some features proposed by Klügel et al.^14^ are also shown in Fig. 4: black lines indicating the fault system around Cumbre Vieja complex, and white lines and filled regions representing the dike system and magma pockets down to about a depth of 20 km.

## Discussion

In the north of the island, the resistive body (> 10^3^ Ω⋅m) extending beneath the Taburiente caldera from sea level to a depth of 10 km (Figs. 2, 3) can be interpreted as the resistive oldest basal complex, consisting of Pliocene seamount and plutonic intrusions^12,14^. This result is supported by the spatial correlation with the dense (> 3000 kg/m^3^ ^35^) intrusive body^13,35–37^. In particular, the high-density anomalies reported at 2 km depth by Prieto et al.^35^ (painted using the white-pointed texture in Fig. 2b) are spatially coincident with our high-resistivity anomaly at the same depth. Such a correlation is also visible in the vertical N–S section, where our high-resistivity anomaly results located inside the limits of the intrusive complex reported by Gonzalez et al.^36^ (painted in white in Fig. 3).

The most interesting features from the 3D resistivity model are located in the southern part of the island, beneath Cumbre Vieja volcanic ridge, where a high resistivity at surface along the mountain ridge is evident, with two conductive layers at greater depths. The resistivity values (< 10 Ω⋅m) are compatible with the signature of a clay alteration cap (smectite and illite-smectite^18^) due to the possible presence of a hydrothermal system around Cumbre Vieja volcano. Such a result can be supported by recent temperature measurements carried out in the south of the island^16^, with values of 90–130 °C indicative of the presence of a clay cap at the top of an active geothermal system^38^. Furthermore, similar features have been recently evidenced in the nearby Tenerife island^24^, and interpreted as the clay cap overlaying the magmatic reservoir^39^.

Another similarity with Tenerife volcanic geothermal system (and with other geothermal areas such as Taupo Volcanic Zone in New Zealand^40^) is the presence of a medium to high resistivity body at depth in the central part of Cumbre Vieja, beneath the two low-resistivity anomalies (Figs. 2, 4). This feature could be related to the hotter part of the geothermal system^40,41^, represented either by a high temperature fluid-filled fractured zone or by the upper part of a shallow intrusive magmatic complex^14^. Although this latter case would suggest a decrease in resistivity that has been not observed in our model, taking into account the reservoir model proposed by Klügel et al.^14^, consisting of small magma pockets instead of a single big magma chamber, we cannot exclude the presence of hot dikes located at a depth of 5–12 km.

These results are supported by the presence of low-density anomalies (~ 2,000 kg/m^3^)^13,35–37^. In particular, in Fig. 2b, the low-density anomalies reported at 2 km by Prieto et al.^35^ are painted using the black-pointed texture; it is noticeable the good spatial correlation at both the sides of the Cumbre Vieja volcanic system. Note that, the spatial coexistence of low-resistivity structures and low-density anomalies can be considered as a proxy for the presence of hydrothermal alteration products^1,24^.

It is important to notice that some authors^13,35–37^ interpreted the presence of a low density anomaly along the western flank of Cumbre Vieja to be due to the presence of unconsolidated debris from the avalanche 560 ka ago on the western flank of Cumbre Nueva volcanic complex, involving a volume exceeding 200 km^3^ and extending to the south of the island^42^. In such a case, the western flank of the Cumbre Vieja complex would have built up filling the embayment, and a sedimentary unit may be present at the base of the Cumbre Vieja lavas, increasing the instability of the volcano^43^. The presence of such a debris layer in the western flank of the volcano has being also supported by a previous MT survey^31^, even if the authors does not exclude the presence of an alteration zone caused by hydrothermal fluid flow. Nevertheless, the presence of invading seawater as the only cause giving rise to the conductive anomaly around the western side of Cumbre Vieja can be discarded because there is no evidence of aquifer salinization in the south of the island and the permeability of the top-surface lavas does not enable a deep penetration of seawater^31,44^.

## Conclusion

A 3D resistivity model of La Palma island (Spain) was obtained from a 44-site MT survey. The model images volcanic structures in the interior of the island. A resistive body in the north, extending to a depth of ~ 10 km has been interpreted as the signature of the Pliocene seamount/plutonic intrusions related to ancient volcanism of the island, and supported by high density anomalies reported in literature. In the south, beneath the active Cumbre Vieja volcanic ridge, a system of two low resistivity zones located above a higher resistivity core has been imaged. Such low-resistivity structures have values compatible with clay alteration caps (illite and illite–smectite) and, due to the co-existence with low-density anomalies reported in literature, they can be considered as a possible signature of hydrothermal alteration products.

## Methods

The time series were processed by employing a robust remote-reference code^33^, yielding mostly stable and high-quality estimates of impedances. The final impedance tensor data quality is good in the period range 10^–3^–10^0^ s, for higher periods the quality becomes site-specific because of noise. Nevertheless, in many sites data are good up to 10^3^ s.

Dimensionality analysis employing the phase tensor method of Caldwell et al.^45^ was performed on our dataset, evidencing a 3D behavior of the resistivity distribution over the island (see Supplementary Figs. S2 and S3).

The fits of model and data for all the measurement sites are reported in Supplementary Fig. S4.

The initial normalized RMS was 20 and the final one 1.97 after 91 iterations using and error floor of 5% for the off-diagonal components and a 10% error floor for the diagonal components. The final error floor distribution for each site is reported in Supplementary Fig. S5.

Two more vertical sections down to a depth of 24 km are reported in Supplementary Figs. S6 and S7: A N–S section (x = 226,000 UTM), and an E–W section (y = 3,181,000 UTM), respectively.

## Supplementary information

Supplementary Information.
