# Supplementary material for: La Palma island (Spain) geothermal system revealed by 3D magnetotelluric data inversion
Source: Sci Rep. 2020 Oct 23;10:18181. doi: 10.1038/s41598-020-75001-z (PMC7585421; doi:10.1038/s41598-020-75001-z)
Supplement: Supplementary file 1 — Supplementary Information. [file 41598_2020_75001_MOESM1_ESM.docx]

**Supplementary information**

**La Palma island (Spain) geothermal system revealed by 3D magnetotelluric data inversion**

**Federico Di Paolo^1*#^, Juanjo Ledo^1,2^, Katarzyna Ślęzak^1**^, David Martínez van Dorth^1^, Iván Cabrera-Pérez^1^, Nemesio M. Pérez^1,3,4^**

^1^Instituto Volcanológico de Canarias (INVOLCAN), San Cristóbal de La Laguna, 38200, Spain.

^2^Departament de Dinàmica de la Terra i de l’Oceà, Universitat de Barcelona, Barcelona, 08028, Spain.

^3^Instituto Tecnológico y de Energías Renovables (ITER), Granadilla de Abona, 38600, Spain.

^4^Agencia Insular de la Energía de Tenerife (AIET), Granadilla de Abona, 38600, Spain.

*now at Dipartimento di Scienze e Tecnologie, Università degli Studi di Napoli “Parthenope”, Naples, 80143, Italy

**now at Departamento de Geofísica, Facultad de Ciencias Físicas y Matemáticas, Universidad de Chile, Santiago, Chile

*Corresponding author: Federico Di Paolo [federico.dipaolo@uniparthenope.it](mailto:federico.dipaolo@uniparthenope.it)

**Methods**

**Data acquisition**


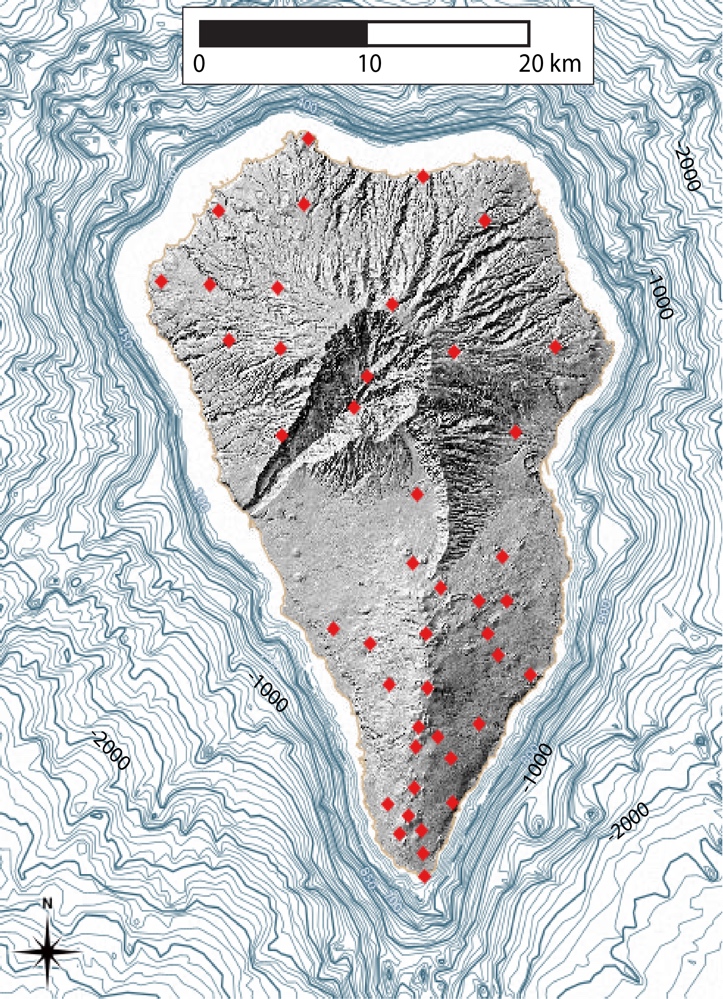


**Figure S1.** Shaded topography of La Palma and bathymetry with a spacing of 50 m (<https://visor.grafcan.es/>). Red diamonds represent the location of the MT sites.

**Data processing**

The magnetotelluric method (MT) is a passive geophysical technique based on the simultaneous measurement of the time variations of the electric and magnetic fields induced in the subsoil by different external sources^46^. The MT has revealed an optimal tool to explore geothermal reservoirs, map the presence of fluids and image significant structural contrasts in the subsoil because the electrical resistivity is a quantity sensitive to the presence of fluids/brines, salts, and, for a vast range of materials, it is also temperature-dependent^1,18^.

In the MT, conductivity distribution within the Earth is determined using relations between components of the electromagnetic variation field measured at the Earth’s surface. The relationship between horizontal components of the electric ($E$) and magnetic ($H$) fields defines the 2×2 complex impedance tensor $\boldsymbol{Z}$ at each frequency as^46^:

$\left[ \begin{matrix} E_{x} \\ E_{y} \end{matrix} \right]=\left[ \begin{matrix} Z_{xx} & Z_{xy} \\ Z_{yx} & Z_{yy} \end{matrix} \right]\left[ \begin{matrix} H_{x} \\ H_{y} \end{matrix} \right]$ (1)

and depends only on the distribution of electrical resistivity in the subsurface^47^.

Scalar impedance components are usually displayed with a pair of real parameters, namely the apparent resistivity ($\rho_{a}$) and phase ($\varphi$), respectively:

$\rho_{a}=\frac{1}{\mu\omega}\left| \boldsymbol{Z} \right|^{2}$ (2)

and

$\varphi=arg(\boldsymbol{Z})$, (3)

with $\mu$ and $\omega$ being the magnetic permeability and the angular frequency, respectively.

Both apparent resistivity and phase depend on frequency, location and Earth’s resistivity structure. They are routinely applied to study the electrical resistivity of the Earth.

Before the inversion of the impedance tensor, dimensionality and strike analysis employing the phase tensor method are needed to better understand the behavior of the resistivity distribution over the island^46^. According to Caldwell *et al*.^45^, the phase tensor ($\boldsymbol{\Phi)}$, that is independent of galvanic distortions, may be represented graphically as an ellipse with the principal axes ($\Phi_{min}$ and $\Phi_{max}$) showing the major and minor axes of the tensor. Note that the phase tensor is a function of the impedance tensor^46^:

$\boldsymbol{\Phi}=\left[ \begin{matrix} \Phi_{xx} & \Phi_{xy} \\ \Phi_{yx} & \Phi_{yy} \end{matrix} \right]=\boldsymbol{P}^{-1}\cdot\boldsymbol{Q}$ (4)

with $\boldsymbol{P}=\mathrm{Re}\left( \boldsymbol{Z} \right)$ and $\boldsymbol{Q}=\mathrm{Im}\left( \boldsymbol{Z} \right)$, respectively. The phase tensor is characterized by rotational invariants, whose features might give important information about the dimensionality of the conductivity structures^45,46^. It can be shown that the phase tensor skew angle ($\beta$), defined in terms of $\boldsymbol{\Phi}$ as:

$\beta=0.5\arctan\left( \frac{\Phi_{xy}-\Phi_{yx}}{\Phi_{xx}+\Phi_{yy}} \right)$ (5)

equals 0 in 1‐D and 2‐D environments. Therefore, from the mapping of $\beta$ at different frequencies, it is possible to infer the dimensionality behavior of the resistivity of the subsoil.

**Dimensionality analysis**

Dimensionality analysis employing the phase tensor method of Caldwell *et al*.^45^ was performed on our dataset, and is reported in Figure S2 in form of a map of skew angle values for each measurement point and for 7 different periods. The values of the skew angle show a 3D behavior of the resistivity distribution over the island.

In order to better show the dimensionality of data, the values reported in Figure S2 have been transformed following the threshold criteria on the skew angle and eccentricity^48,49^ by using the MTpy code^50,51^. In Figure S3, the evaluated dimensionality of data for each point is clearer and a 3D behavior of the data emerges, especially at long periods, implying the need for a complete 3D inversion to be performed.

**
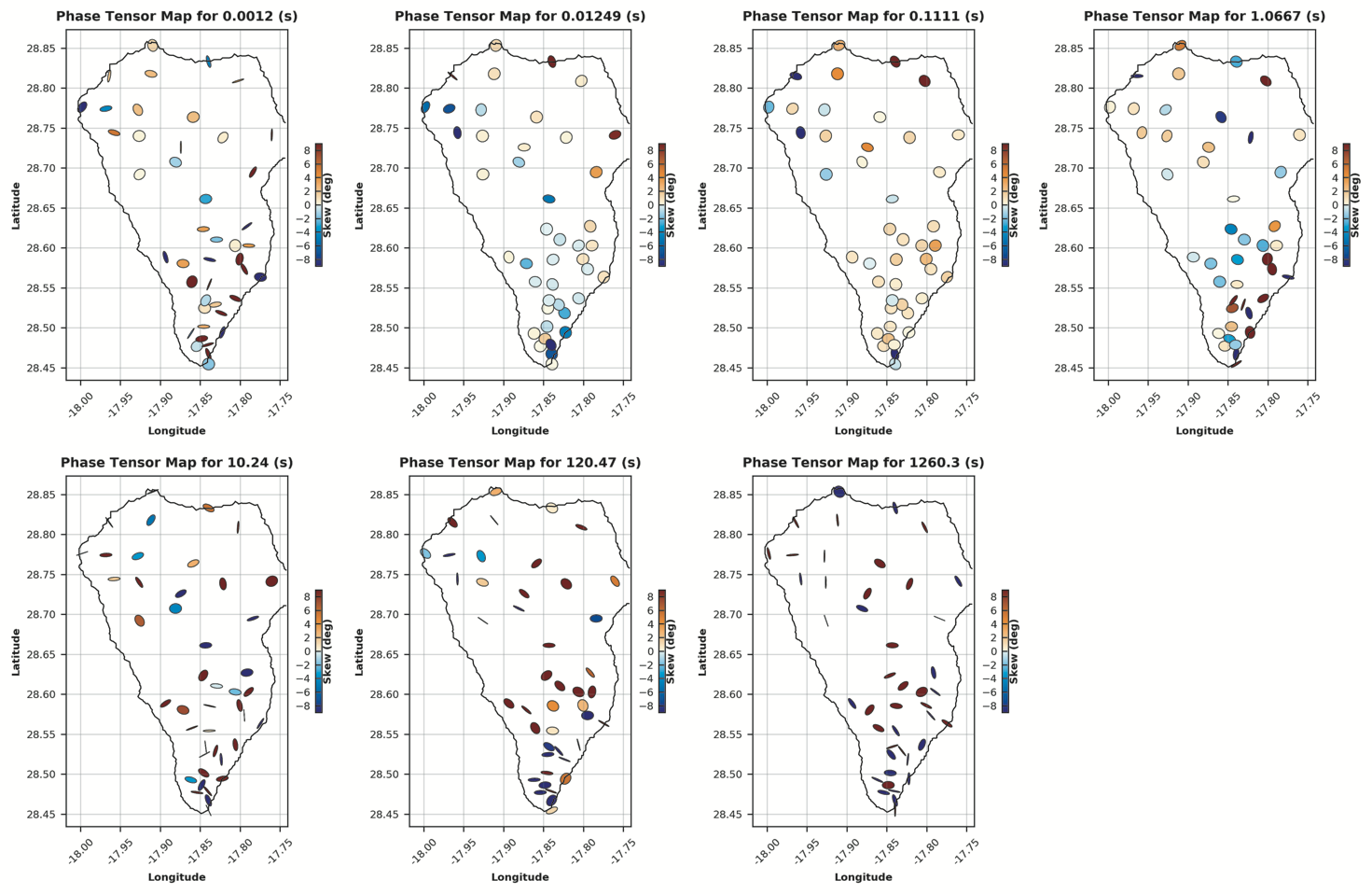
**

**Figure S2.** Skew angle ellipses maps for seven different periods evaluated employing the phase tensor method of Caldwell *et al*.^45^.

**
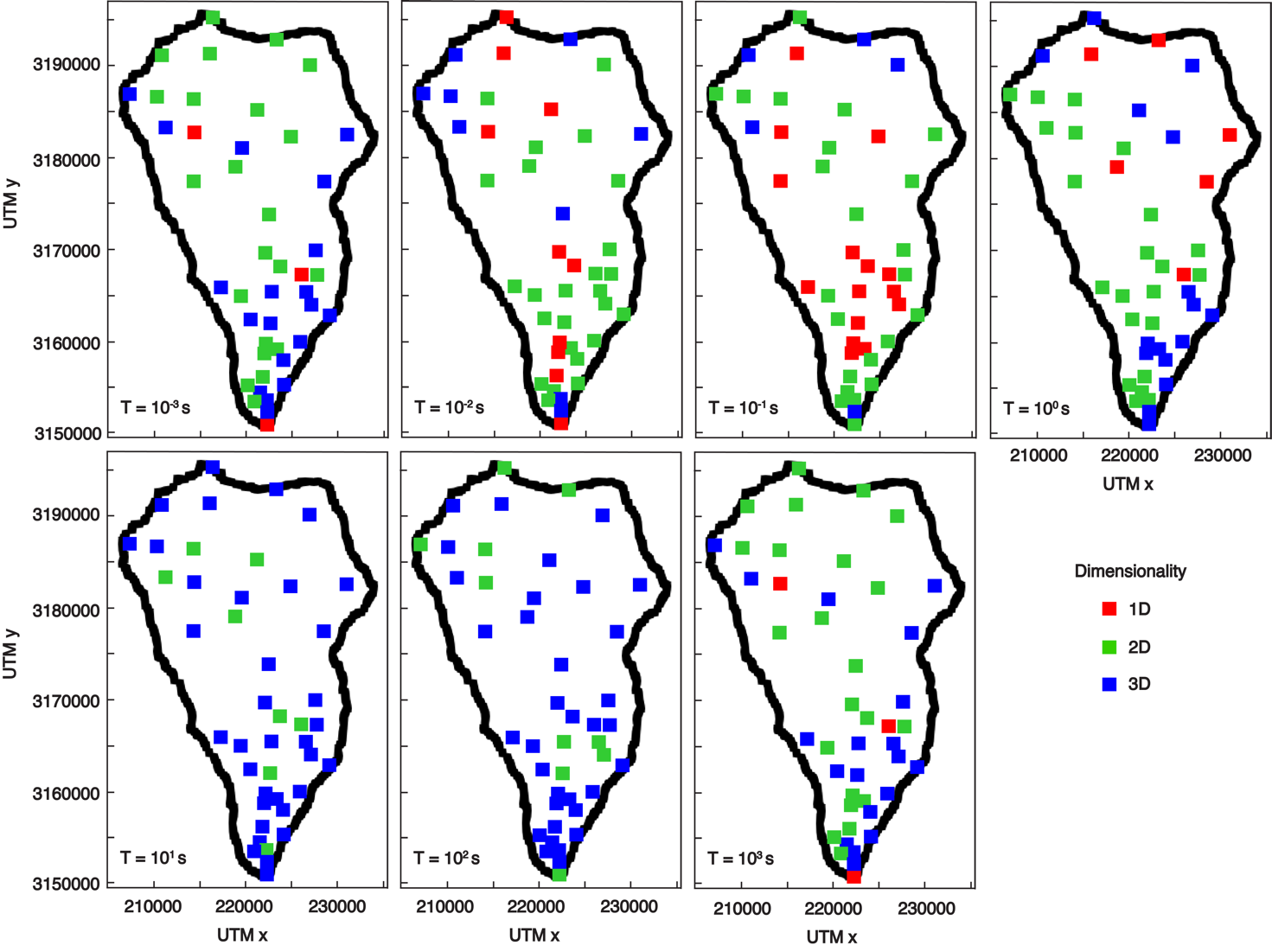
**

**Figure S3.** Dimensionality maps for seven different periods evaluated from the phase tensor following the threshold criteria on the skew angle and eccentricity^48,49^ using the MTpy code^50,51^.

**MT modelling**

The time series were processed by employing a robust remote-reference code^33^, yielding mostly stable and high-quality estimates of impedances. The final impedance tensor data quality is good in the period range 10^-3^-10^0^ s, for higher periods the quality becomes site-specific because of noise. Nevertheless, in many sites data are good up to 10^3^ s.


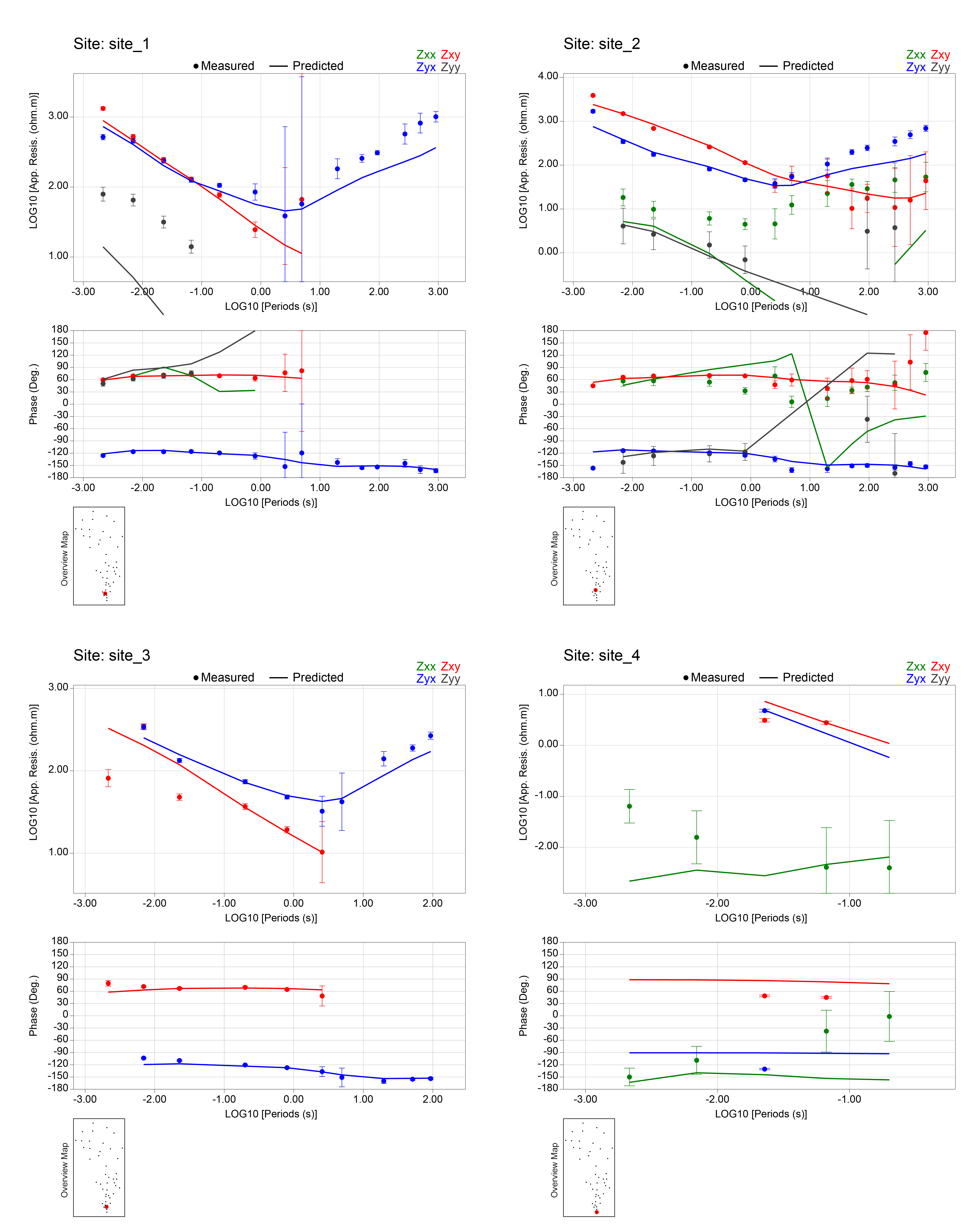


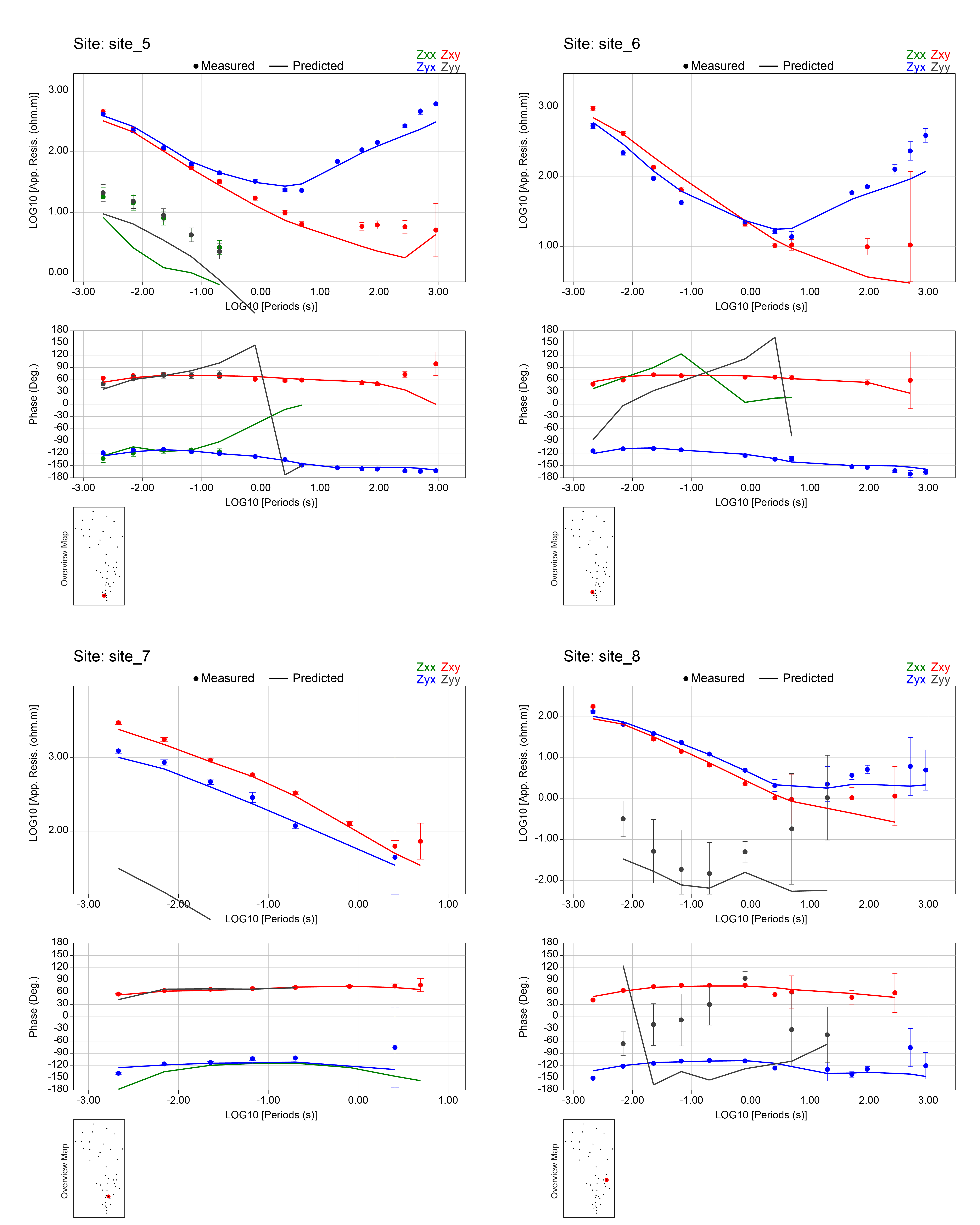


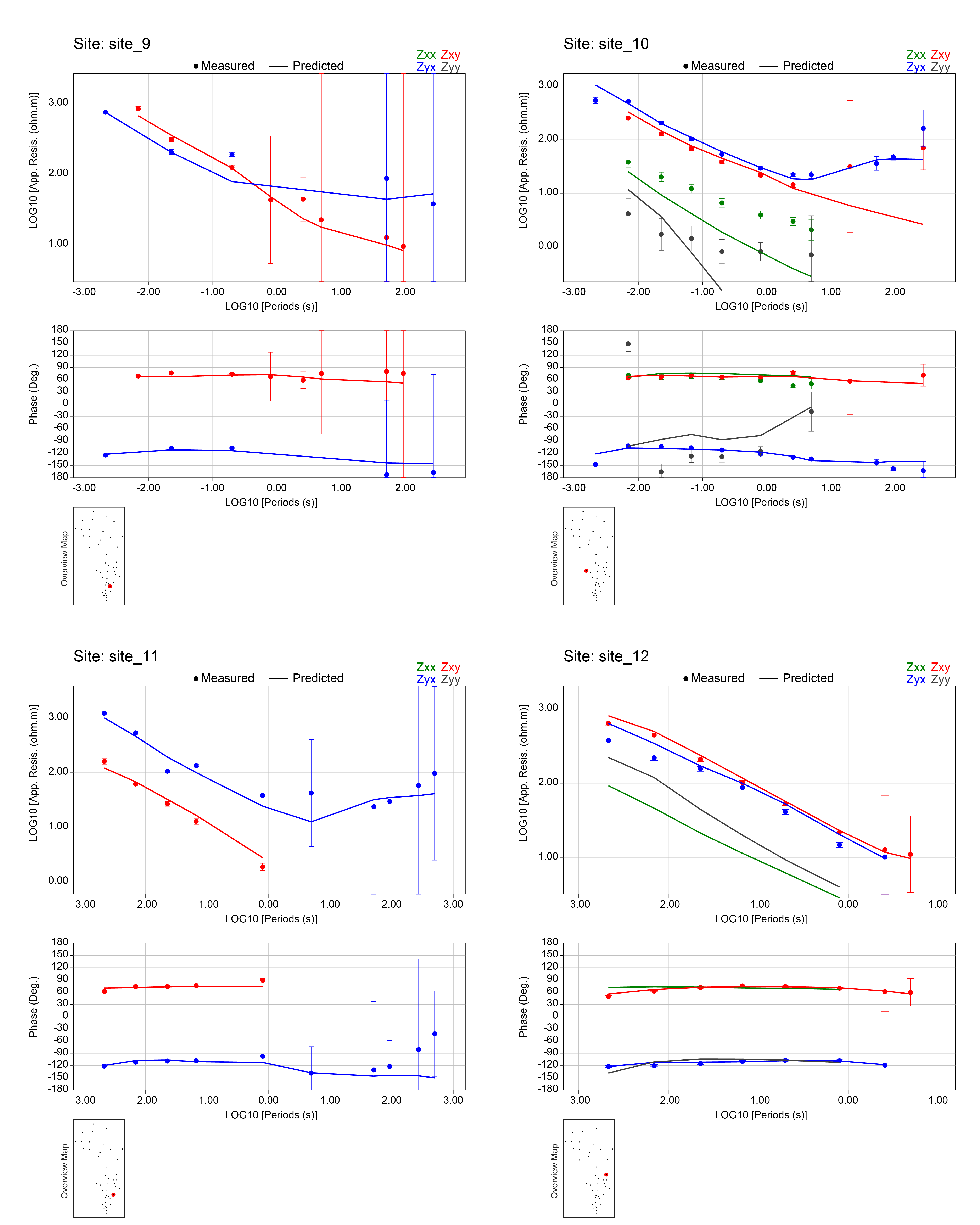


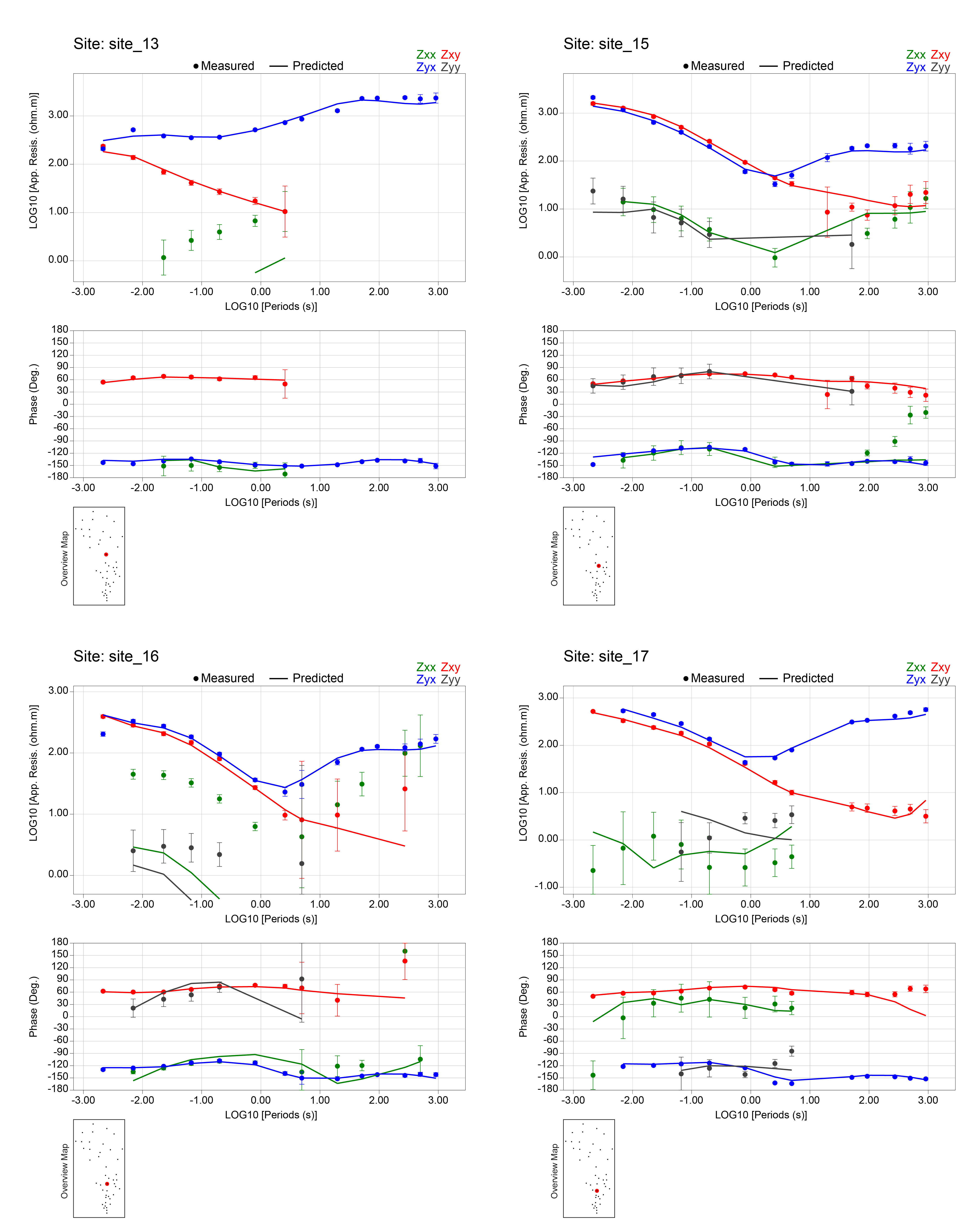


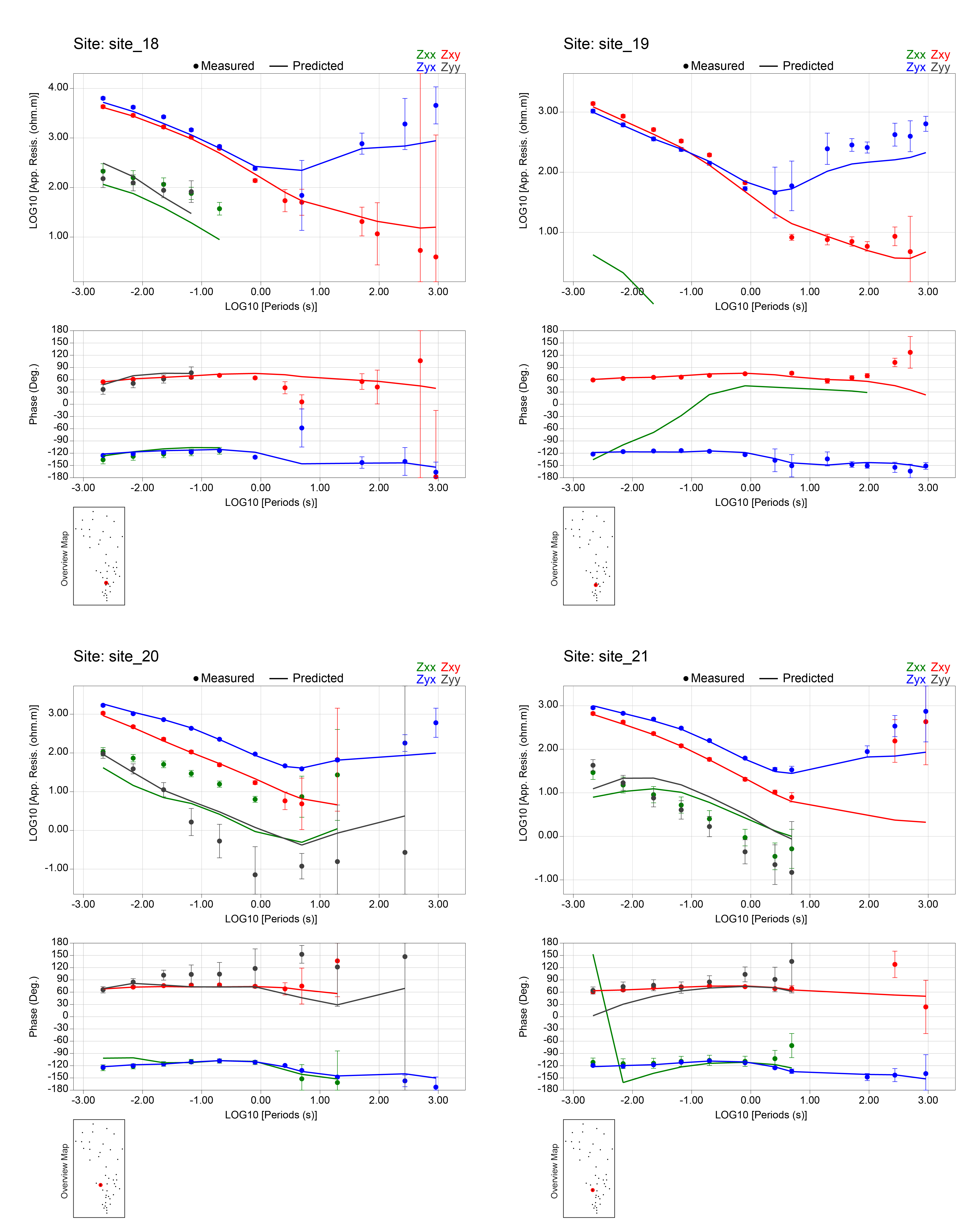


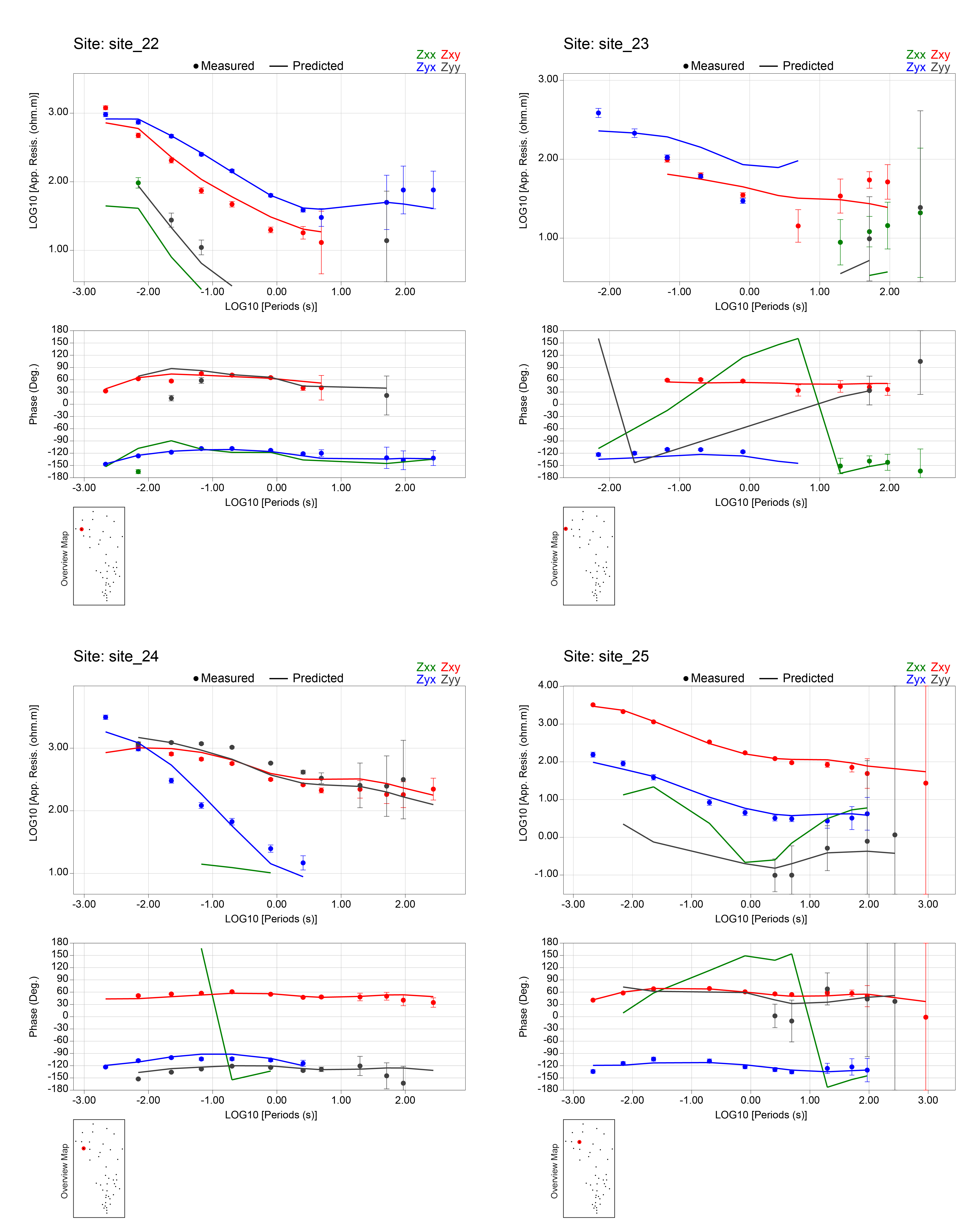


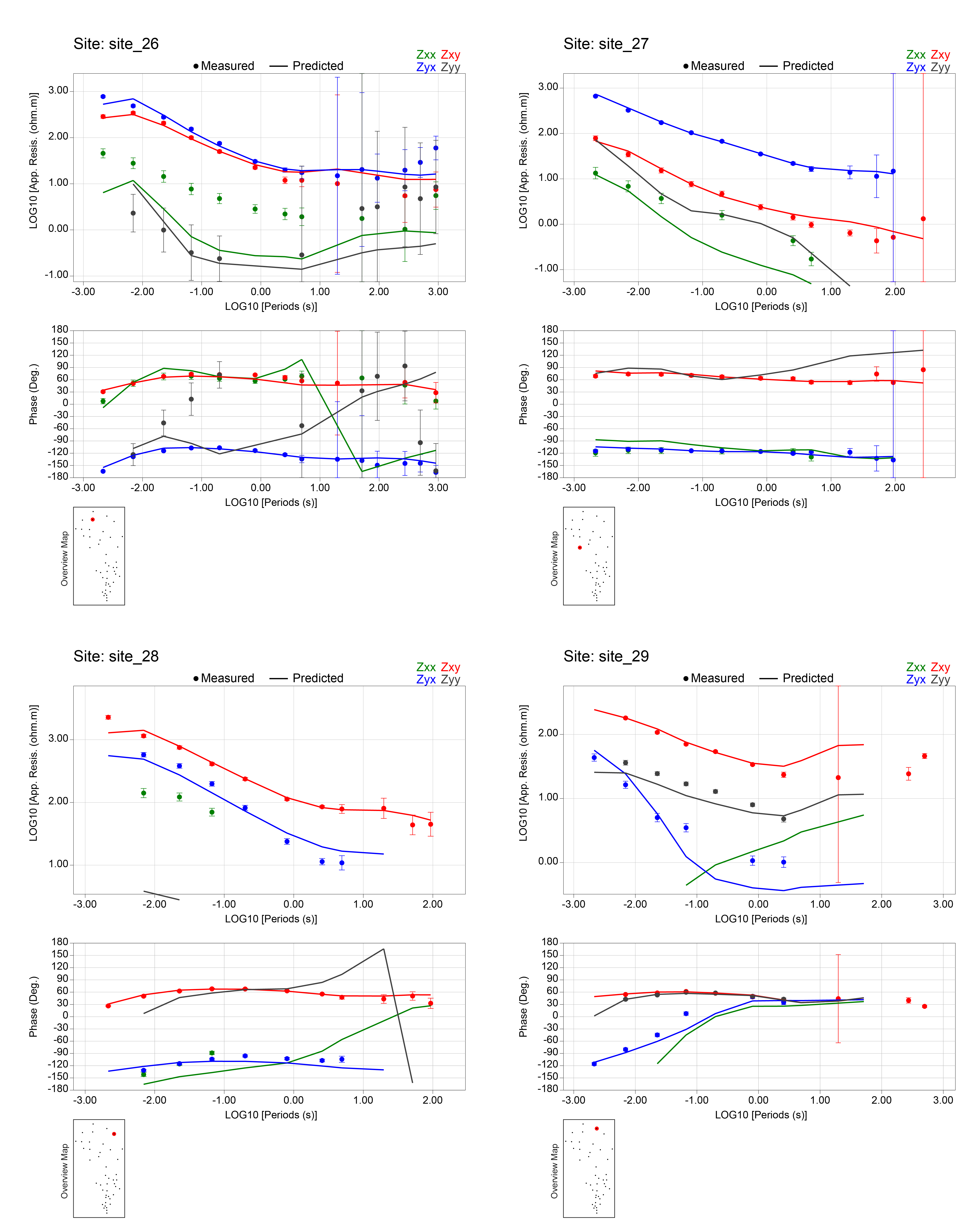


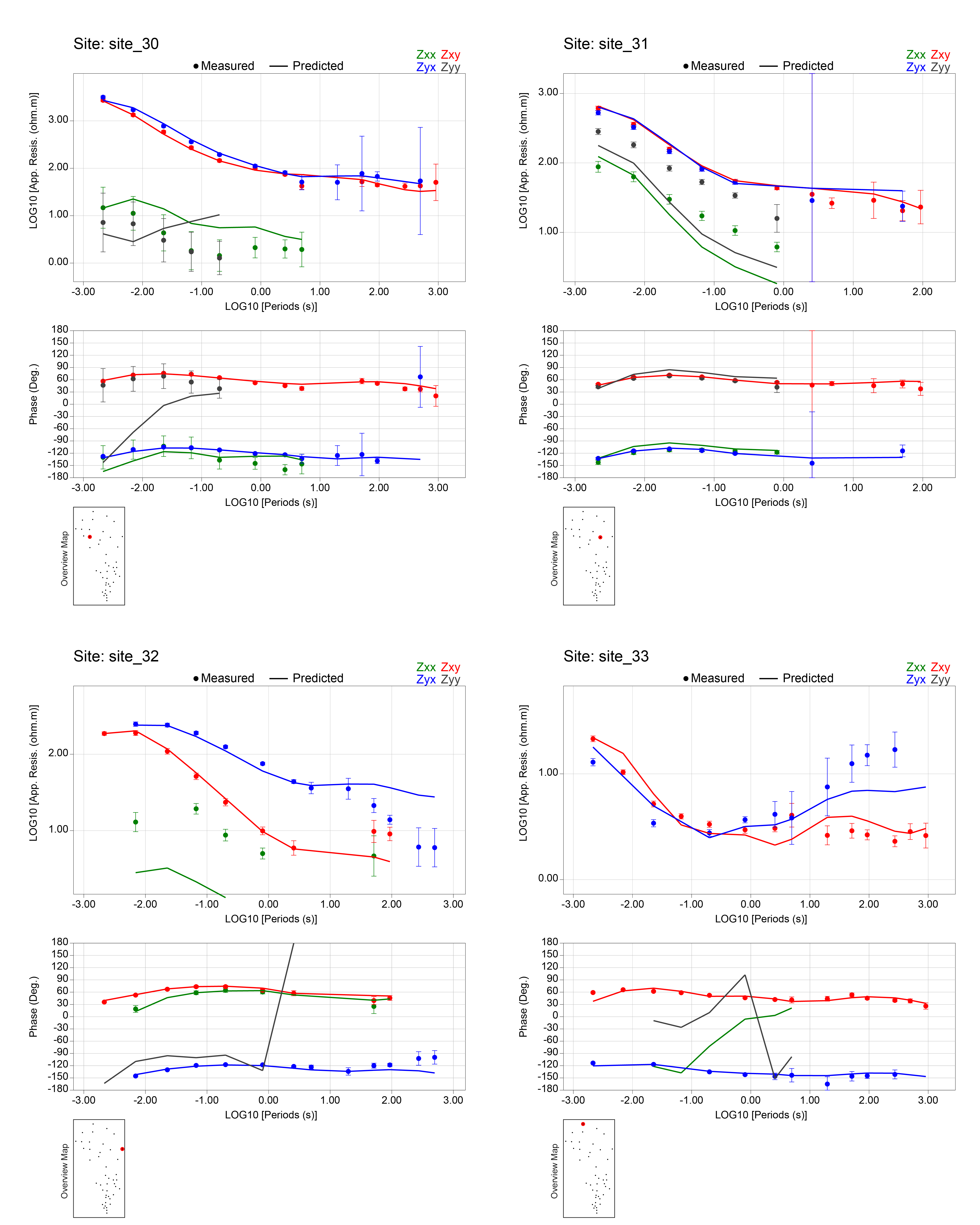


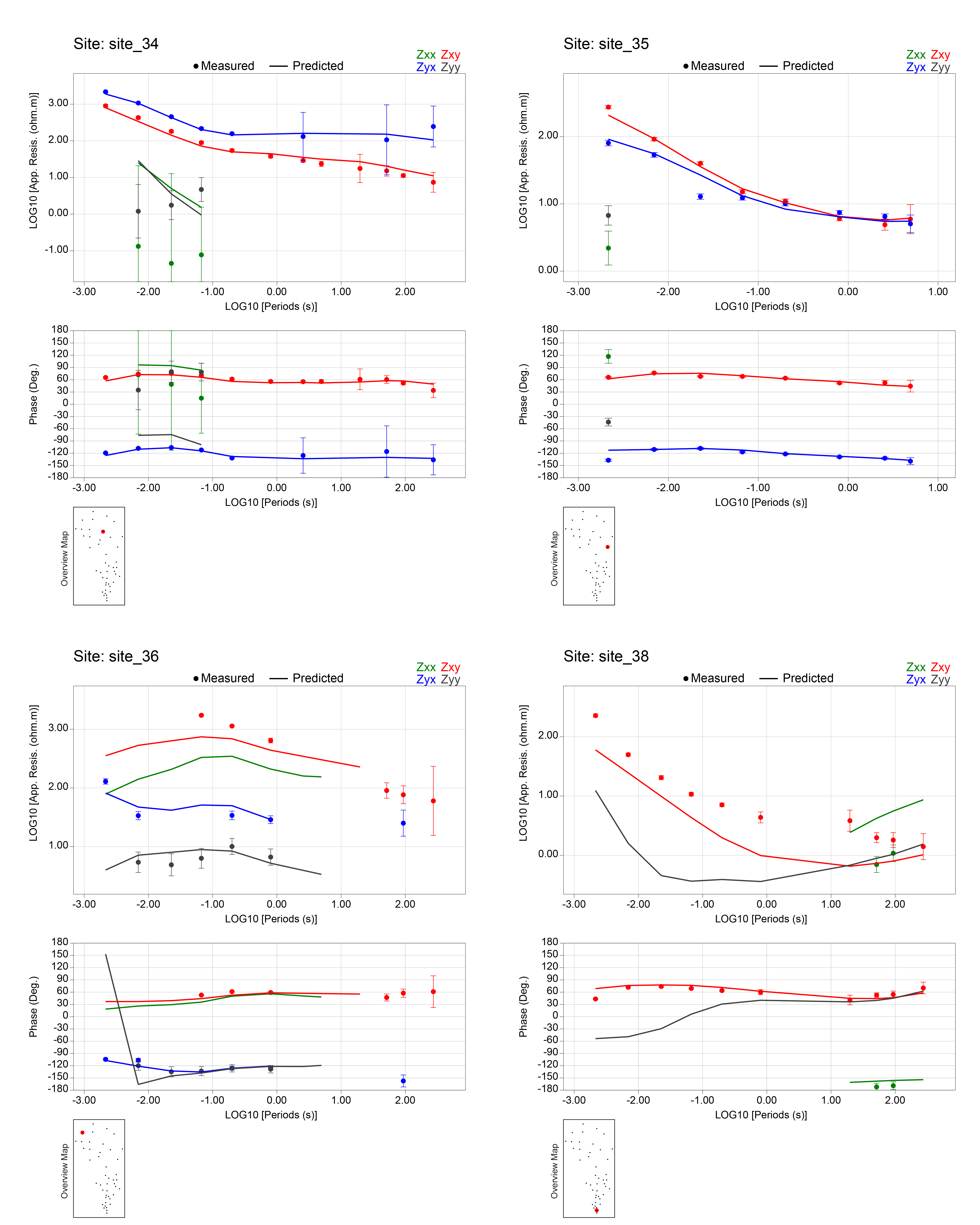


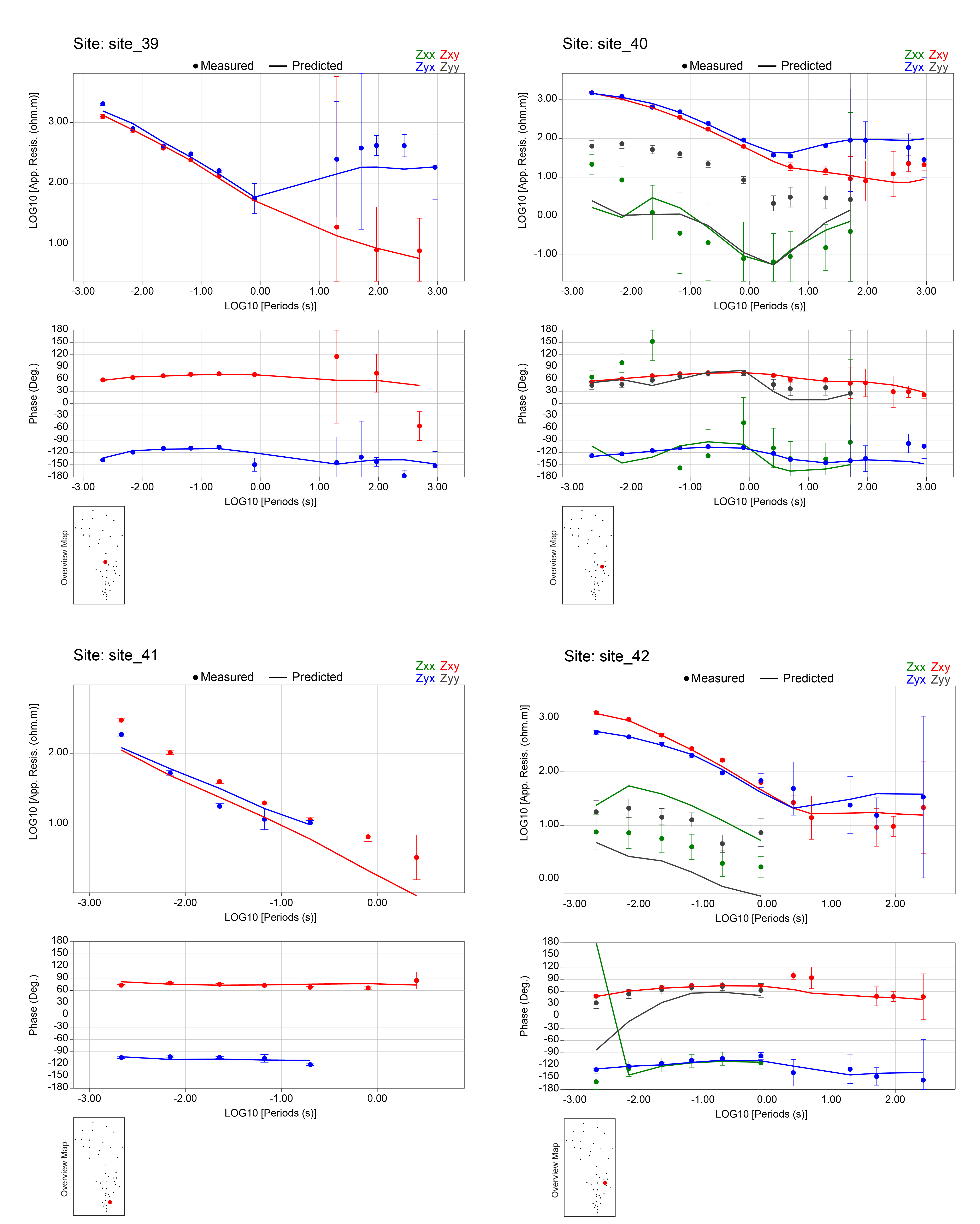


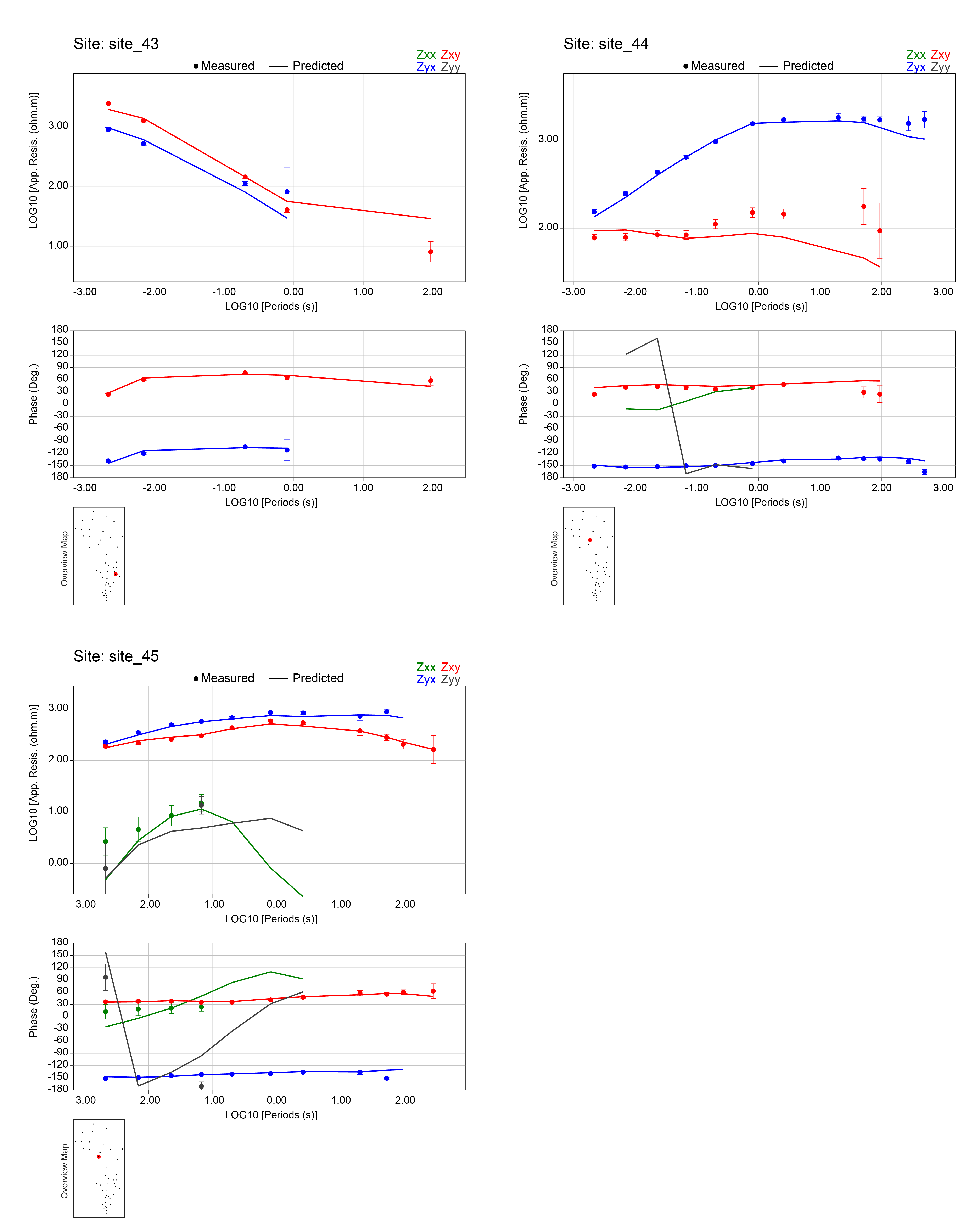


**Figure S4.** Original and fitted data as a function of period for the entire dataset.

**Error distribution on the inversion**

The initial normalized RMS was 20 and the final one 1.97 after 91 iterations using and error floor of 5% for the off-diagonal components and a 10% error floor for the diagonal components. Figure S5 show the error floor distribution for each site.


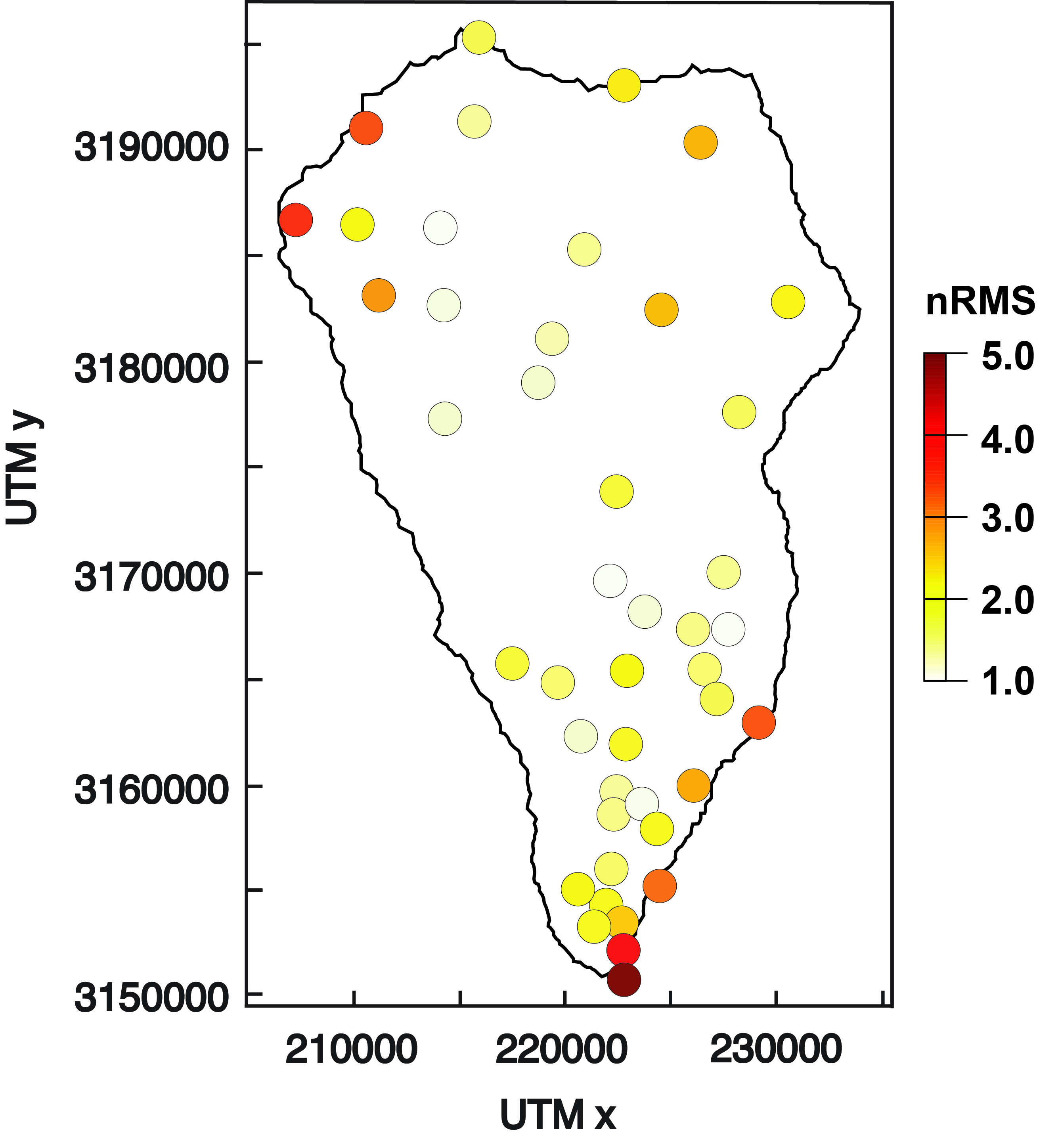


**Figure S5.** RMS after 91 iterations at each MT station (circles).

**Results**


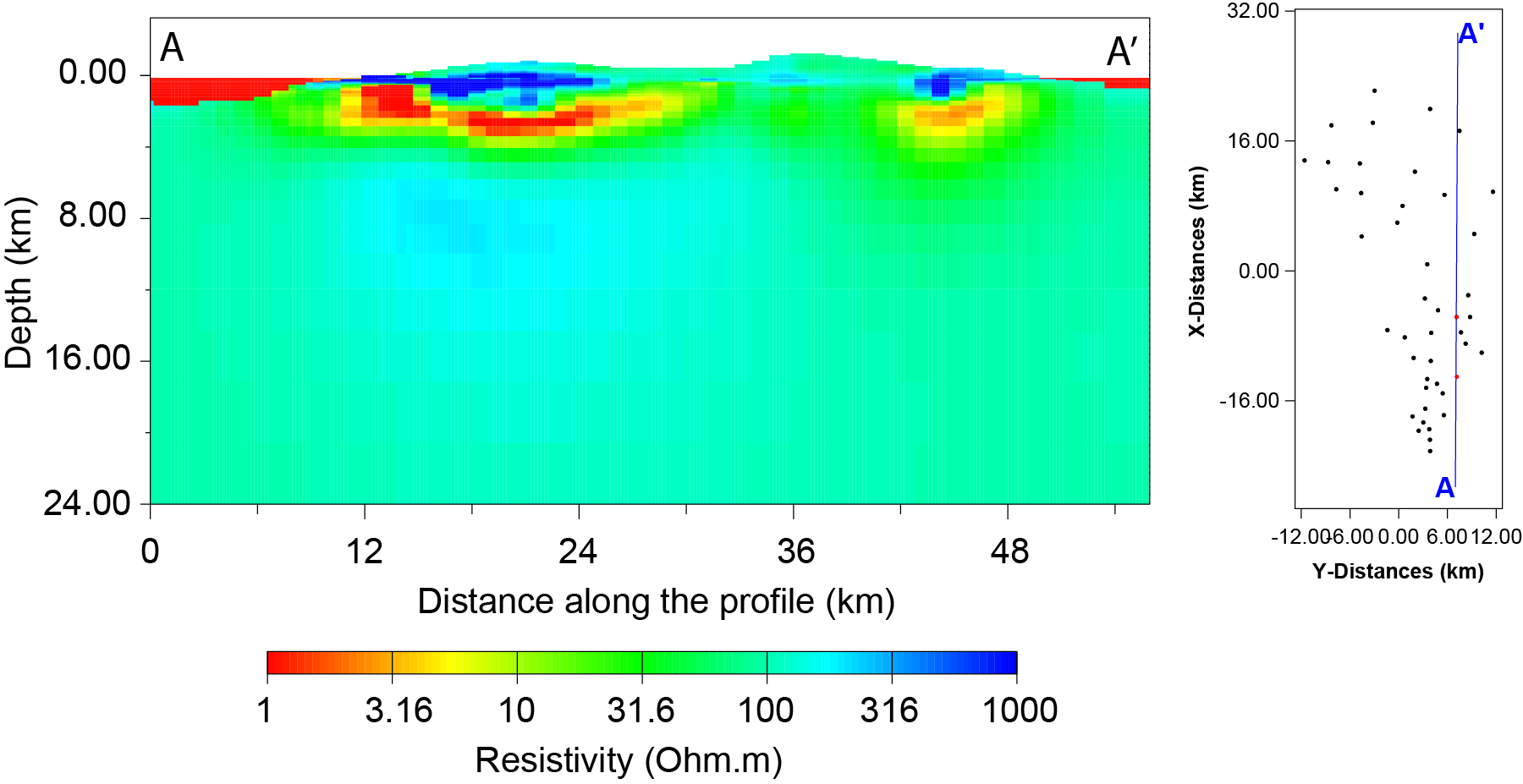


**Figure S6.** Vertical North-South section (x = 226000 UTM) from the 3D resistivity model.

**
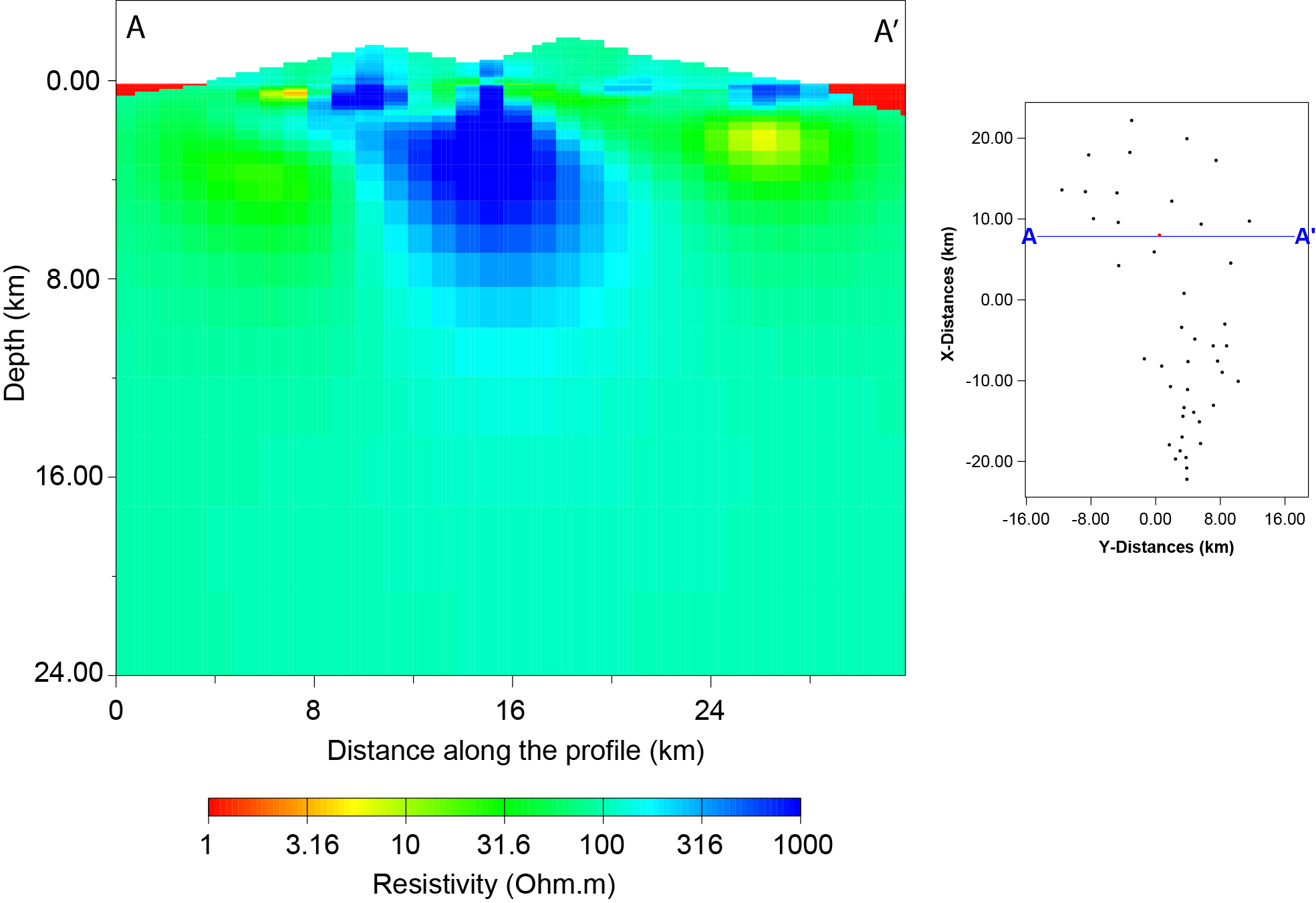
**

**Figure S7.** Vertical East-West section (y = 3181000 UTM) from the 3D resistivity model.

**References**

**1.** Spichak, V., & Manzella, A. Electromagnetic sounding of geothermal zones. *J. Appl. Geophys.,* **68**(4), 459-478 (2009).

**2.** IRENA. Geothermal Power: Technology Brief. International Renewable Energy Agency, Abu Dhabi, (2017).

**3.** Staudigel, H., & Schmincke, H. U. The pliocene seamount series of La Palma/Canary Islands. *J. Geophys. Res.*, **89**(B13), 11195-11215 (1984).

**4.** Anguita, F., & Hernán, F. A propagating fracture model versus a hot spot origin for the Canary Islands. *Earth Planet. Sci. Lett*., **27**(1), 11-19 (1975).

**5.** Araña, V., & Ortiz, R. The Canary Islands: Tectonics, magmatism and geodynamic framework. in *Magmatism in extensional structural settings* (eds. Kampunzu, A. B., and Lubala R. T.), 209-249 (Springer, 1991).

**6.** Pérez, N. M. 3He/4He isotopic ratios in volcanic-hydrothermal discharges from the Canary Islands, Spain: implications on the origin of the volcanic activity. *Mineral. Mag*., **58**, 709-710 (1994).

**7.** Carracedo, J. C. *et al*. Hotspot volcanism close to a passive continental margin: the Canary Islands. *Geol. Mag*., **135**(5), 591-604 (1998).

**8.** Dañobeitia, J. J., & Canales, J. P. Magmatic underplating in the Canary Archipelago. *J. Volcanol. Geotherm. Res.*, **103**(1-4), 27-41 (2000).

**9.** Anguita, F., & Hernán, F. The Canary Islands origin: a unifying model. *J. Volcanol. Geotherm. Res.*, **103**(1-4), 1-26 (2000).

**10.** Carracedo, J. C., Rodriguez-Badiola, E., Guillou, H., Nuez Pestana, J. D. L., & Pérez Torrado, F. J. Geology and volcanology of La Palma and El Hierro, Western Canaries. *Estud. Geol*., **57**, 175-273 (2001).

**11.** Troll, V. R., & Carracedo, J. C. *The geology of the Canary Islands*. (Elsevier, 2016).

**12.** Galipp, K., Klügel, A., & Hansteen, T. H. Changing depths of magma fractionation and stagnation during the evolution of an oceanic island volcano: La Palma (Canary Islands). *J. Volcanol. Geotherm. Res.*, **155**(3-4), 285-306 (2006).

**13.** Camacho, A. G. *et al*. Structural results for La Palma island using 3‐D gravity inversion. *J. Geophys. Res*., **114**(B5) (2009).

**14.** Klügel, A., Hansteen, T. H., & Galipp, K. Magma storage and underplating beneath Cumbre Vieja volcano, la Palma (Canary Islands). *Earth Planet. Sci. Lett*., **236**(1-2), 211-226 (2005).

**15.** Padrón, E. *et al*. Helium emission at Cumbre Vieja volcano, La Palma, Canary Islands. *Chem. Geol*., **312**, 138-147 (2012).

**16.** Padrón, E. *et al*. Dynamics of diffuse carbon dioxide emissions from Cumbre Vieja volcano, La Palma, Canary Islands. *Bullet. Volcanol.*, **77**(4), 28 (2015).

**17.** Soler, C. (2007). *La historia de la Fuente Santa. Santa Cruz de Tenerife*. (Turquesa, 2007).

**18.** Muñoz, G. Exploring for geothermal resources with electromagnetic methods. *Surv. Geophys*., **35**(1), 101-112 (2014).

**19.** Uchida, T., & Sasaki, Y. Stable 3D inversion of MT data and its application to geothermal exploration. *Explor. Geophys*., **37**(3), 223-230 (2006).

**20.** Heise, W., Caldwell, T. G., Bibby, H. M., & Bannister, S. C. Three-dimensional modelling of magnetotelluric data from the Rotokawa geothermal field, Taupo Volcanic Zone, New Zealand. *Geophys. J. Int.,* **173**(2), 740-750 (2008).

**21.** Newman, G. A., Gasperikova, E., Hoversten, G. M., & Wannamaker, P. E. Three-dimensional magnetotelluric characterization of the Coso geothermal field. *Geothermics*, **37**(4), 369-399 (2008).

**22**. Bertrand, E. *et al*. Magnetotelluric imaging of upper‐crustal convection plumes beneath the Taupo Volcanic Zone, New Zealand. *Geophys. Res. Lett*., **39**(2) (2012).

**23.** Kelbert, A., Egbert, G. D., & deGroot-Hedlin, C. Crust and upper mantle electrical conductivity beneath the Yellowstone Hotspot Track. *Geology*, **40**(5), 447-450 (2012).

**24.** Piña-Varas, P. *et al*. 3-D magnetotelluric exploration of Tenerife geothermal system (Canary Islands, Spain). *Surv. Geophys*., **35**(4), 1045-1064 (2014).

**25.** Coppo, N. *et al*. 3-D magnetotelluric investigations for geothermal exploration in Martinique (Lesser Antilles). in *Proceedings of the World Geothermal Congress*, 1501.06541 (2015).

**26.** Piña‐Varas, P. *et al*. Vertical collapse origin of Las Cañadas caldera (Tenerife, Canary Islands) revealed by 3‐D magnetotelluric inversion. *Geophys. Res. Lett.*, **42**(6), 1710-1716 (2015).

**27.** Peacock, J. R., Mangan, M. T., McPhee, D., & Wannamaker, P. E. Three‐dimensional electrical resistivity model of the hydrothermal system in Long Valley Caldera, California, from magnetotellurics. *Geophys. Res. Lett*., **43**(15), 7953-7962 (2016).

**28.** García-Yeguas, A. *et al*. A 3D joint interpretation of magnetotelluric and seismic tomographic models: The case of the volcanic island of Tenerife. *Comput. Geosci.*, 109, 95-105 (2017).

**29.** Samrock, F., Grayver, A. V., Eysteinsson, H., & Saar, M. O. Magnetotelluric image of transcrustal magmatic system beneath the Tulu Moye geothermal prospect in the Ethiopian Rift. *Geophys. Res. Lett*., **45**(23), 12-847 (2018).

**30.** Maithya, J., & Fujimitsu, Y. Analysis and interpretation of magnetotelluric data in characterization of geothermal resource in Eburru geothermal field, Kenya. *Geothermics*, **81**, 12-31 (2019).

**31.** García, X., & Jones, A. G. Internal structure of the western flank of the Cumbre Vieja volcano, La Palma, Canary Islands, from land magnetotelluric imaging*. J. Geophys. Res*., **115**(B7) (2010).

**32.** Gamble, T. D., Goubau, W. M., & Clarke, J. Error analysis for remote reference magnetotellurics. *Geophysics*, **44**(5), 959-968 (1979).

**33.** Egbert, G. D., & Booker, J. R. Robust estimation of geomagnetic transfer functions. *Geophys. J. Int.*, **87**(1), 173-194 (1986).

**34.** Egbert, G. D., & Kelbert, A. Computational recipes for electromagnetic inverse problems. *Geophys. J. Int.*, **189**(1), 251-267 (2012).

**35.** Prieto, J. F. *et al*. Geodetic and Structural Research in La Palma, Canary Islands, Spain: 1992–2007 Results. *Pure Appl. Geophys*, **166**(8-9), 1461-1484 (2009).

**36.** González, P. J., Tiampo, K. F., Camacho, A. G., & Fernández, J. (2010). Shallow flank deformation at Cumbre Vieja volcano (Canary Islands): Implications on the stability of steep-sided volcano flanks at oceanic islands. *Earth Planet. Sci. Lett.*, **297**(3-4), 545-557.

**37.** Fernández, J. *et al*. DInSAR, GPS and gravity observation results in La Palma, Canary Islands. in *Proceedings of the 2008 IEEE Second Workshop on Use of Remote Sensing Techniques for Monitoring Volcanoes and Seismogenic Areas*, 1-5 (2008).

**38.** Browne, P.R.L. Hydrothermal alteration in active geothermal fields. *Annu. Rev. Earth Planet. Sci.*, **6**, 229-250 (1978).

**39.** Piña-Varas, P., Ledo, J., Queralt, P., Marcuello, A., & Perez, N. On the detectability of Teide volcano magma chambers (Tenerife, Canary Islands) with magnetotelluric data. *Earth Planets Space*, **70**(1), 1-14 (2018).

**40.** Heise, W., Caldwell, T. G., Bibby, H. M., & Bannister, S. C. Three-dimensional modelling of magnetotelluric data from the Rotokawa geothermal field, Taupo Volcanic Zone, New Zealand. *Geophys. J. Int.*, **173**(2), 740-750 (2008).

**41.** Ussher, G., Harvey, C., Johnstone, R., & Anderson, E. Understanding the resistivities observed in geothermal systems. in *Proceedings of the World Geothermal Congress*, 1915-1920 (2000).

**42.** Carracedo, J. C., Day, S. J., Guillou, H., & Torrado, F. J. P. Giant quaternary landslides in the evolution of La Palma and El Hierro, Canary Islands. *J. Volcanol. Geotherm. Res.*, **94**(1-4), 169-190 (1999).

**43.** Day, S. J., Carracedo, J. C., Guillou, H., & Gravestock, P. Recent structural evolution of the Cumbre Vieja volcano, La Palma, Canary Islands: volcanic rift zone reconfiguration as a precursor to volcano flank instability? *J. Volcanol. Geotherm. Res.*, 94(1-4), 135-167 (1999).

**44.** Consejo Insular de Aguas de La Palma. Plan Hidrológico de La Palma, *Bol. Off. Canarias*, **237** (2018).

**45.** Caldwell, T. G., Bibby, H. M., & Brown, C. The magnetotelluric phase tensor. *Geophys. J. Int.*, **158**(2), 457-469 (2004).

**46.** Chave, A. D., & Jones, A. G. (Eds.). *The magnetotelluric method: Theory and practice*. Cambridge University Press (2012).

**47.** Vozoff, K. The magnetotelluric method. in *Electromagnetic Methods in Applied Geophysics: Volume 2, Application, Parts A and B*, 641-712 (Society of Exploration Geophysicists, 1991).

**48.** Bibby, H. M., Caldwell, T. G., & Brown, C. Determinable and non-determinable parameters of galvanic distortion in magnetotellurics. *Geophys. J. Int.*, **163**(3), 915-930 (2005).

**49.** Booker, J. R. The magnetotelluric phase tensor: a critical review. *Surv. Geophys*., **35**(1), 7-40.

**50.** Krieger, L., & Peacock, J. R. MTpy: A Python toolbox for magnetotellurics. *Comput. Geosci.*, **72**, 167-175 (2014).

**51.** Kirkby, A., Zhang, F., Peacock, J., Hassan, R., & Duan, J. The MTPy software package for magnetotelluric data analysis and visualisation. *J. Open Source Softw.*, **4**(37), 1358 (2019).
